# Supplementary material for: Super-resolution orbital angular momentum holography
Source: Nat Commun. 2023 Apr 4;14:1869. doi: 10.1038/s41467-023-37594-7 (PMC10073211; doi:10.1038/s41467-023-37594-7)
Supplement: Supplementary file 1 — Supplementary Information [file 41467_2023_37594_MOESM1_ESM.pdf]

Supplementary Information

Super-resolution orbital angular momentum holography

Zijian Shi[1,2,3], Zhensong Wan[1,2,3], Ziyu Zhan[1,2,3], Kaige Liu[1,2,3], Qiang Liu[1,2,3,*], and Xing Fu[1,2,3,*]

[1]Department of Precision Instrument, Tsinghua University, Beijing 100084, China
[2]State Key Laboratory of Precision Space-time Information Sensing Technology (Tsinghua University), Beijing 100084, China
[3]Key Laboratory of Photonic Control Technology (Tsinghua University), Ministry of Education, Beijing 100084, China

*Corresponding author. Email: qiangliu@tsinghua.edu.cn; fuxing@tsinghua.edu.cn

Supplementary Note 1: Characterization of the resolution of the reconstructed image OAM holography

Under the paraxial approximation, the Fourier transform holographic system (Fig. S1) has the following relationship:

$$\frac{M}{2a}=\frac{\sin(\theta)}{\lambda}=\frac{u_{\max}}{2\lambda f} \tag{1}$$

where M refers to the number of discrete points on the hologram plane, a is the size of holographic plane, θ is the maximum diffraction angle of the diffracted light, u_{max} is the maximum coordinate of the image plane, and f represents the focal length of Fourier transform lens. Thus, the maximum coordinate of image plane can be deduced from Equations 1 as

$$u_{\max}=\frac{M\lambda f}{a} \tag{2}$$

Since the number of discrete points in the holographic plane is equal to the number of discrete points in the image plane, the interval between two discrete points in the image plane is given by

$$\Delta u=\frac{\lambda f}{a} \tag{3}$$

Note that the numerical aperture of the holographic system can be calculated as NA=a/f. Thus, the interval between two discrete points in the image plane is rewritten as

$$\Delta u=\frac{\lambda}{\mathrm{NA}} \tag{4}$$

Since the discrete numerical space and actual physical space have a simple correspondence (Equations 4), we can numerically calculate the OAM pixel size using fast Fourier transformation. The OAM pixel size $d_{\max}$ is defined as the number of discrete points where the intensity drops to 15% of the maximum value. The intensity distribution of OAM pixels on the image plane can be written as

$$I_{OAM}=\left|FT\left[circ(\frac{r}{R})e^{il_j\varphi}\right]\right|^2 \tag{5}$$

For arbitrary value of $\gamma=d_{\max}/L$, the sampling distance of reconstructed image is $L=d_{\max}/\gamma$, and the resolution, which is denoted as the pixel number per inch, is calculated as 1 inch over L. For the example in Fig. 1b in the main text, the largest addressable OAM mode with the helical index l=200 occupies 170 pixels, corresponding to 0.602 mm with the setting that NA is 0.15 and the wavelength is 532 nm. Therefore, we have $L=d_{\max}$=0.602 mm for the case of γ=1, and L=0.128 mm for the case of γ=4.7, while the resolution for the latter case is 198 pixels/inch.

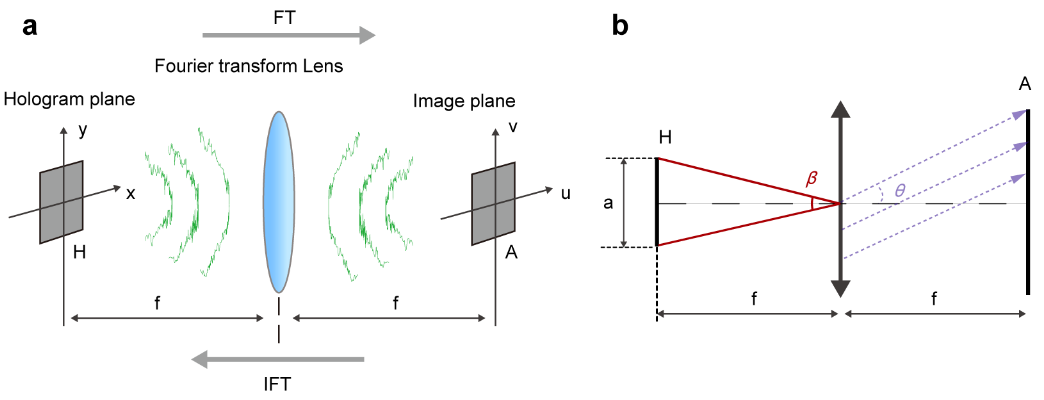

Fig. S1. Schematic diagram of Fourier transform holographic system. a Schematic diagram of Fourier transform holographic system. **b** Light propagation model in the Fourier transform system.

As stated in the main text, the case of γ=1 represents the OAM holography at resolution limit, while the cases of γ>1 represent the super-resolution OAM holography. Table S1 compares the resolution limit in OAM holography and that in a conventional optical system. The main interest of the conventional resolution limit lies at the image plane of the optical system, where the distribution of the point spread function is usually the diffraction pattern of the circular aperture (Bessel function), and the factors affecting the resolution limit are the light wavelength and the numerical aperture (NA) of the optical system. In contrast, the resolution limit in OAM holography is examined at the back focus plane (Fourier plane) of the lens, where the point spread function is the distribution of the maximum addressable OAM mode in the reconstructed image. The resolution limit of OAM holography depends on the helical index of the maximum addressable OAM mode, in addition to the optical wavelength and the NA of the Fourier holographic system (see Fig. S1).

Table S1 Comparison between the resolution limit in a conventional optical system and that in OAM holography.

	**Main location**	**Point spread function**	**Affecting factors**
**Conventional resolution limit**	Image plane of the optical system	The diffraction pattern of the circular aperture	Wavelength and NA
**Resolution limit in OAM holography**	Back focus plane (Fourier plane) of the lens	The distribution of the maximum addressable OAM mode	Wavelength, NA and l_{max}

Supplementary Note 2: Illustration of the temporal multiplexing

Temporal multiplexing is indeed the addition of a phase grating in the temporal domain, and can be interpreted as a superposition of two sub-processes (see Fig. S2). The phase grating generates n incoherent wave vectors in the temporal domain, the number of which is equal to the number of multiplexed holograms. Each wave vector incident on the hologram produces an independent diffraction pattern. The observed reconstructed image consists of m diffraction patterns superimposed in an intensity manner, where m is determined by the duration Δ of each diffraction

pattern and the observation time duration *T*.

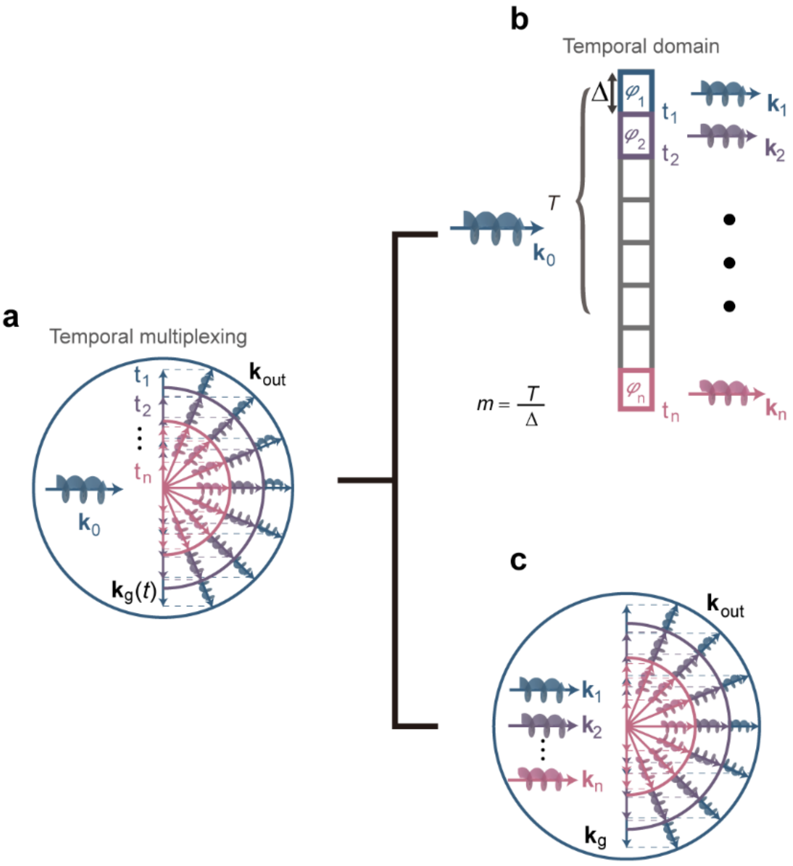

Fig. S2. Illustration of the temporal multiplexing concept. a Coherence suppression with temporal multiplexing. **b** A wave vector is incident on a phase grating in the temporal domain and generates *n* incoherent wave vectors. **c** The *n* incoherent wave vectors produce *n* independent diffraction patterns. It is worth noting that the *n* incoherent wave vectors of Fig. S2c have the same direction with respect to Fig. 2c, because the *n* incoherent wave vectors originate from the temporal domain expansion of the same wave vector.

Supplementary Note 3: The interference between different OAM channels at the same pixel (SPI)

Based on an original image I_j that is an amplitude-only distribution, and a random-phase distributionΦ_{j0}, we can compute a complex-amplitude OAM-multiplexed hologram (CAH) as

$$H^{mul} = \sum_{j=1}^{M} A_j e^{il_j\varphi} \tag{6}$$

where l_j and φ represent the helical mode index and azimuthal angle, respectively, *i* represents the imaginary symbol, *M* denotes the total number of OAM multiplexing channels, while the complex amplitude A_j of each image channel and I_j are related by the Fourier transform as

$$\sum_m \sum_n I_j(x_m, y_n) e^{i\phi_{j0}(x_m, y_n)} \delta(x_m, y_n) = FT\{A_j\} \tag{7}$$

where δ denotes the Dirac functions, *m* and *n* represents the pixel position. The image of OAM

holography projected on a screen is a collection of pixels, or image points. Assuming the illumination by coherent unit-amplitude plane wave carrying OAM, the reconstructed field of an OAM-multiplexed hologram can be expressed as

$$E_{mul} = \sum_{j=1}^{M} \left\{ \sum_{m}\sum_{n} I_j(x_m, y_n) e^{i\phi_{j0}(x_m, y_n)} \delta(x_m, y_n) \otimes FT\left[circ(\frac{r}{R}) e^{il_j\varphi} \right] \right\} \tag{8}$$

where $\otimes$ represents the convolution symbol, R is the radius of hologram, and r is the radius in the polar coordinate system. Now we consider the intensity at a point on the image as

$$I_{mul}(x_1, y_1) = \left| \sum_{j=1}^{M} I_j(x_1, y_1) FT\left[circ(\frac{r}{R}) e^{il_j\varphi} \right] e^{i\phi_{j0}(x_1, y_1)} \right|^2 \tag{9}$$

which indicates that the intensity at each point is not determined by the original image alone, but by the combinative effect of superposed mode pixels and the original image. The uncontrolled interference between different OAM channels leads to an unwanted strong fluctuation of intensity, due to random states of OAM modes and random phases attached to pixels, thereby contaminating the reconstructed images. In contrast, we assume a pseudo incoherent case where the images belonging to different OAM channels are superposed in an intensity manner, as expressed by

$$I_{mul}(x_1, y_1) = \sum_{j=1}^{M} \left| I_j(x_1, y_1) FT\left[circ(\frac{r}{R}) e^{il_j\varphi} \right] \right|^2 \tag{10}$$

which means the intensity at each pixel are stable without uncontrollable interference, separating each image channel from the others. Moreover, a mode-selective aperture array can be used to rule out the reconstructed image channels with doughnut-shaped intensity distributions.

Supplementary Note 4: Intensity fluctuation analysis of reconstructed results with varying multiplexing channel number

To compare the reconstruction qualities between coherent and pseudo incoherent cases, a set of sparsely sampled simple images are encoded into multiple OAM channels respectively, with the helical mode index interval of Δl=1. Specifically, the reconstructed image with the mode index of l=1 is used for quantitative comparison.

Figure S3a describes how the coefficient of variation (CV) of reconstructed images varies with the number of multiplexing channels K. For the coherent case, CV increases from 8.7% at K=5 to 11.8% at K=17 that corresponds to the traditional boundary (γ=1) that is imposed by the sampling criterion (see also Fig. S3b), and then grows more rapidly beyond the boundary, rising up to 29.9% at K=35 (see also Fig. S3c), leading to a seriously degraded reconstruction quality as shown in the left inset of Fig. S3a. In contrast, CV for the pseudo incoherent case remains almost unchanged at 3.4~3.7%, against varying channel numbers for the whole range of $\gamma \leq 2$. Performances with other helical mode intervals (Δl=2, 3, 4, 5) are shown in Fig. S4, implying that the reconstruction quality deterioration at dense sampling can be effectively weakened for the coherent case, by using sufficiently large index interval, at the price of reduced multiplexing capacity.

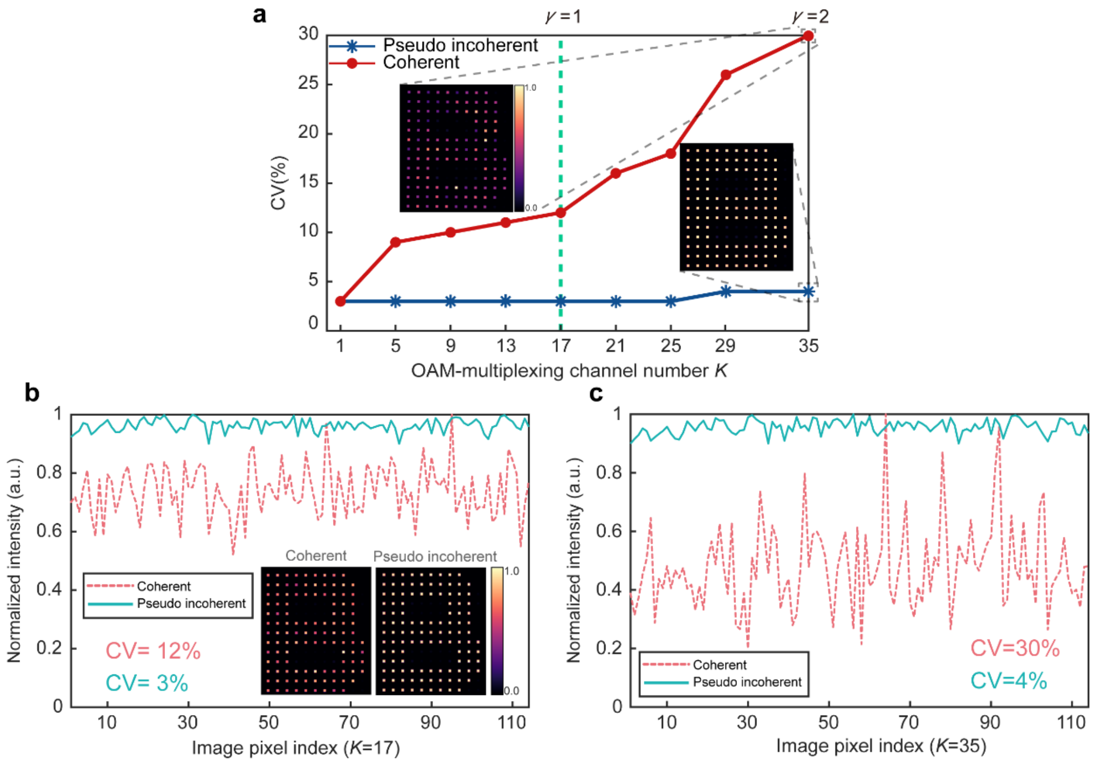

Fig. S3. Intensity fluctuation analysis of reconstructed results with varying multiplexing channel number *K*. a CV of reconstructed images versus *K*. Vertical dashed line: traditional boundary with γ=1. Insets: reconstructed images at γ=2 for pseudo incoherent and coherent cases. **b-c** Normalized intensities of all pixels in the decoded images with *K*=17 (**b**) and *K*=35 (**c**) respectively. Image pixels are sorted in top-to-bottom and left-to-right sequences.

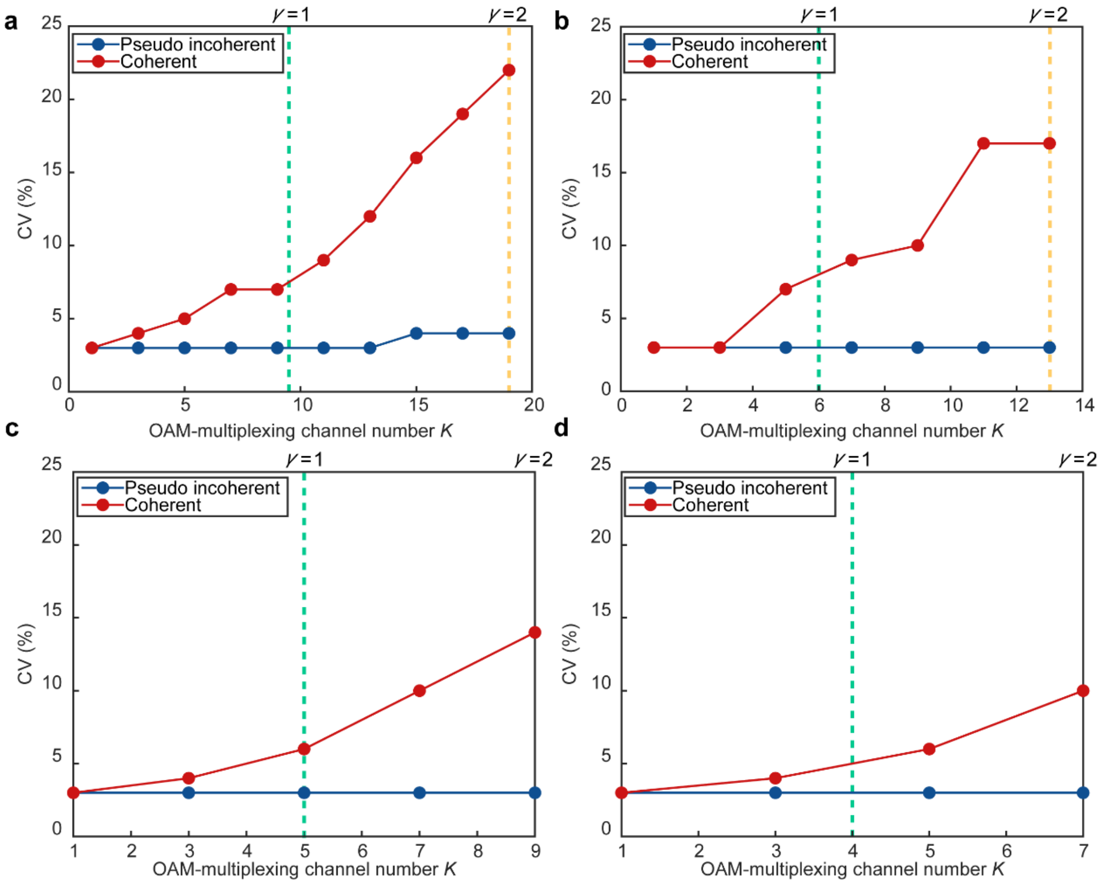

Fig. S4. CV of reconstructed images versus the number of OAM-multiplexing channels. a Δl=2. **b** Δl=3. **c** Δl=4. **d** Δl=5.

Supplementary Note 5: OAM-multiplexed interference model with small index interval

As stated in the main text, in addition to SPI, there exists interference between OAM channels at the pixel and those from adjacent pixels, termed as adjacent pixel interference (API). Here we adjust the helical mode index in Fig. 4 in the main text from l=0, 5, 10, 15 to l=0, 1, 2, 3, which allows the contributions from both SPI and API in the interference model. Similarly, we evaluate the intensity fluctuation at the signal location using the CV (Fig. S5a) and the noise level at the non-signal location using the average intensity (Fig. S5b). For the coherent case, the intensity fluctuation at the signal location reaches 13.4% at γ=1 due to SPI, and degrades violently to 67.9% at γ=2.78 due to the combined effect of SPI and API. In addition, for both coherent and pseudo incoherent cases, the average intensity at the non-signal location increases rapidly beyond γ=2, surpassing 40% of the signal intensity at γ=2.78, which indicates that the background noise represented by the intensity of the non-signal region is not negligible in this case.

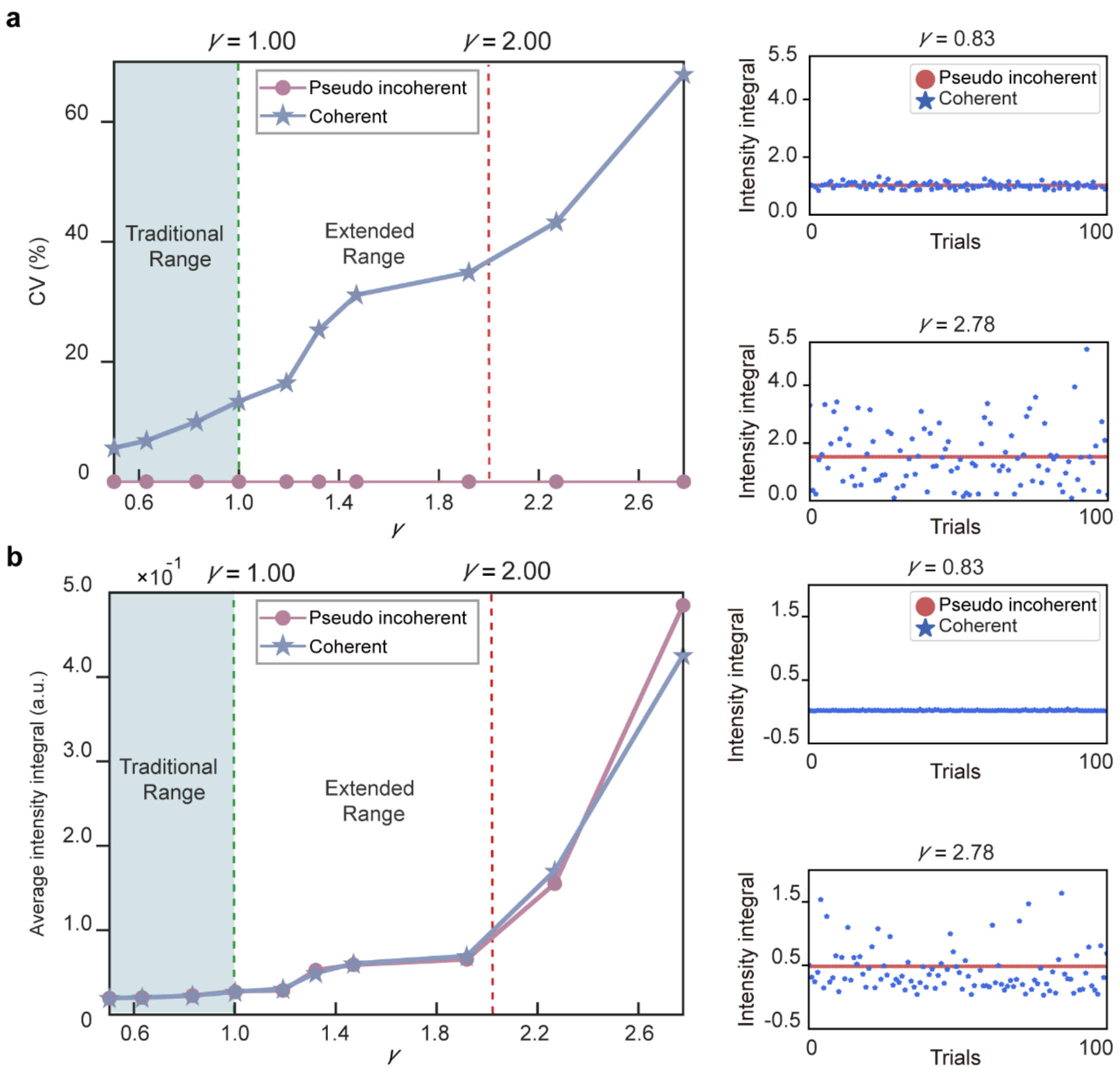

Fig. S5. Interference model with index interval of Δ*l*=1. a Intensity fluctuations of signal location versus *γ*. **b** Average intensity of non-signal location versus *γ*.

Supplementary Note 6: API effect for the spot array with different intensities

To investigate the API effect for the spot array with different intensities, we set random intensity values (uniformly distributed in the range of 0 to 1) for the 8 pixels around the center pixel of interest and run 100 trials to obtain the results as shown in Fig. S6. The case depicted in Fig. S6a is actually the API model when grayscale images are multiplexed. For the coherent case, it shows a slightly smaller level of intensity fluctuation at γ = 2.9 (CV=30%), compared with Fig. 4c with spot array having the same intensity (CV=36%). For the pseudo-incoherent case, CV becomes nonzero, reaching 1% at γ = 2.9, due to the fact that intensities of the pixels around the center one vary at each trial, thereby introducing slight intensity fluctuation. For non-signal location, Fig. S6b shows a decrease in the average intensity of noise at the center pixel (2.5% of signal intensity at γ = 2.9) compared with that in Fig. 4e (6.0% of the signal intensity).

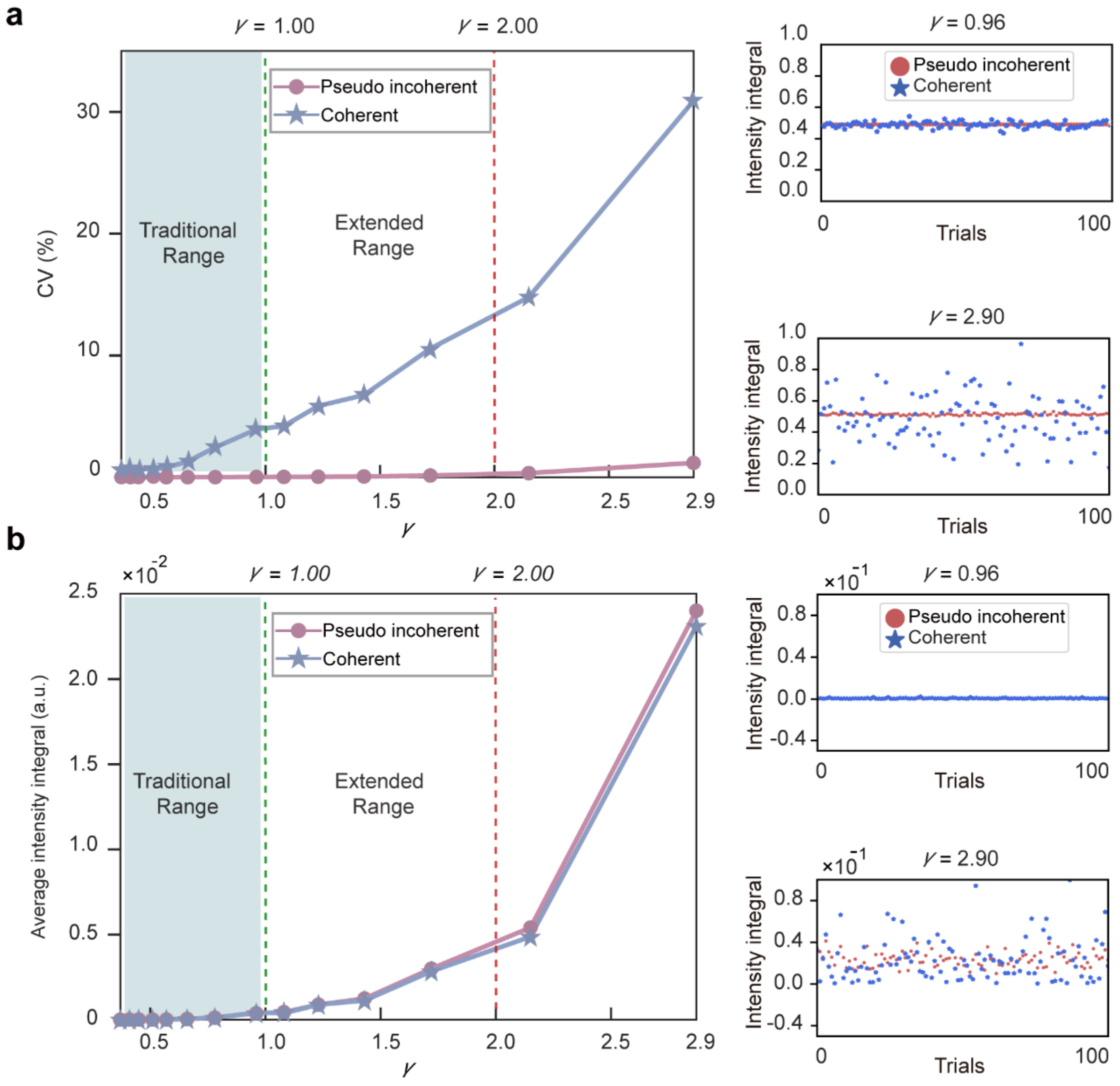

Fig. S6 API model for the spot array with random intensities. a Fluctuations of intensity integral versus γ, for the API effect on a signal location, with densely packed OAM modes (l=0, 5, 10, 15) at nine adjacent pixels. The CV value indicating the intensity fluctuations is calculated from 100 trials. Specific intensity results of each trial are shown for the cases of γ=0.96 and γ=2.90 (right column). **b** Average intensity integral versus γ for the API effect on a non-signal location, with densely packed OAM modes (l=5, 10, 15) at nine adjacent pixels.

Supplementary Note 7: SPI and API models of temporal multiplexing

Temporal multiplexing is a coherent means of approximating the pseudo-coherent case, and its degree of approximation is related to the number of temporal multiplexing holograms. For the case of temporal multiplexing of 50 holograms, we investigate the models of SPI and API. In Figs. S7 and S8, we compare the temporal multiplexing case with the coherent case in terms of both SPI and API effects. Comparing the results of Fig. S7 with those in Fig. 3, it can be concluded that the temporal multiplexing case and the pseudo-incoherent case behave consistently in the model of SPI. The intensity fluctuations of reconstructed images for the temporal multiplexing case shown in Fig. S7c are almost the same as that for the pseudo-incoherent case. In addition, the intensity distributions of three representative pixels in the reconstructed image for the temporal

multiplexing case (Fig. S7b, S7c) are extremely close to the pseudo-incoherent case (Fig. 3b, 3c). The capability of temporal multiplexing of suppressing intensity fluctuations is illustrated in Fig. S8a, where the CV of temporal multiplexing rises to 5% under super-resolution conditions of γ = 2.9, while the CV of pseudo-incoherent case is always close to zero (Fig. 4c).

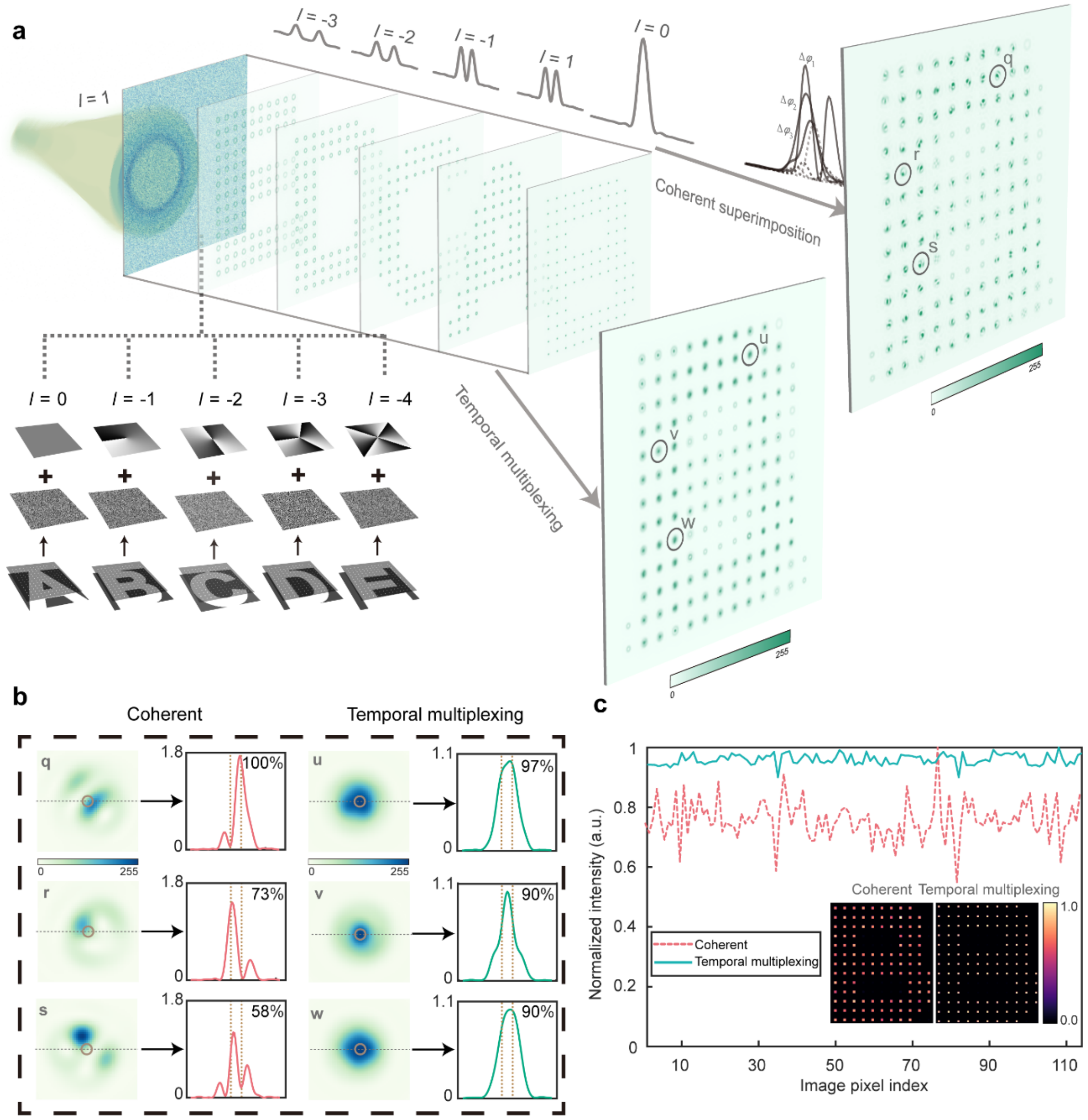

Fig. S7 SPI model of the case of temporal multiplexing of 50 holograms. a OAM beams with the helical mode index *l*=1 is incident on the OAM-multiplexed hologram, and images carried by different OAM channels are displayed simultaneously (left). Performances of coherent case (top right), and temporal multiplexing case (bottom right) are compared. **b** Three representative pixels from the reconstructed image in **a**, for the coherent (left) and temporal multiplexing (right) cases. Intensity distributions are presented in both 2D profiles and 1D cross-sectional views where the values indicate the normalized intensity integral within the region as denoted by vertical dotted lines. **c** Intensities of all pixels in the post-processed results of the reconstructed image in **a**.

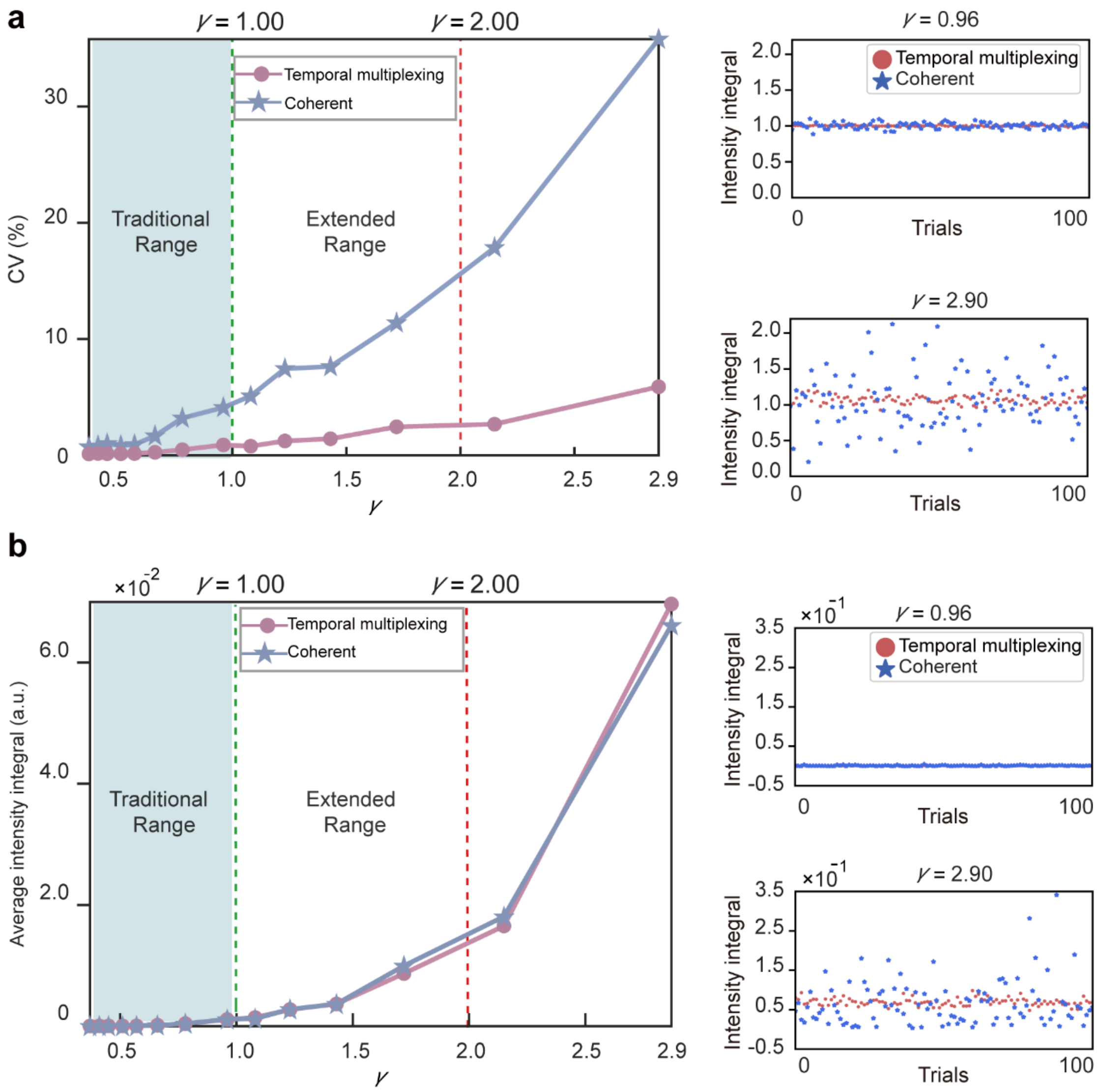

Fig. S8 API model of the case of temporal multiplexing of 50 holograms. a Fluctuations of intensity integral versus γ, for the API effect on a signal location, with densely packed OAM modes (l=0, 5, 10, 15) at nine adjacent pixels. The CV value indicating the intensity fluctuations is calculated from 100 trials. Specific intensity results of each trial are shown for the cases of γ=0.96 and γ=2.90 (right column). **b** Average intensity integral versus γ for the API effect on a non-signal location, with densely packed OAM modes (l=5, 10, 15) at nine adjacent pixels.

Supplementary Note 8: Characterization of two types of interference in reconstructed images

We study the combinative effect of SPI and API, by comparing three types of OAM multiplexing holograms as shown in Fig. S9. The first type is SPI dominant, as already manifested in Fig. 3 in the main text, having sparse sampling interval of L=30 λ/NA that represents low-resolution images, and small helical mode index interval of Δl=1 (l=0, -1, -2, -3, -4), corresponding to γ=0.4. The second type is API dominant, as studied in Fig. 4 in the main text, with a denser sampling interval of L=15 λ/NA but a large index interval of Δl=5 (l=0, -5, -10, -15, -20), corresponding to γ=2.5. The third type has both densely sampled target images (L=5 λ/NA, equivalently high-resolution) and small index interval (l=0, -1, -2, -3, -4), having γ=2.5 as well.

The reconstructed images of three types of holograms in the coherent case are shown in Fig. S9a, where the post-processed results after a mode-selective aperture array are shown at the bottom row, illustrating that the SPI and API effects in the reconstructed images of OAM holography induce strong crosstalk in the post-processed results. For the first type (left column), the SPI patterns are distributed at all pixels, while for the second type (middle column), it is API that causes undesired intensity fluctuations at the signal location. Notably, for the reconstructed image of third type (right column) that is filled with heavy speckle noise, we stress that there are two reasons for the dramatically aggravated crosstalk level, compared with the second type. On one hand, obviously the SPI due to small index interval of Δl=1 is overlaid with API. On the other hand, despite that both types have the same value of γ, API itself becomes more severe for the third type, because the outermost helical mode has a much smaller index ($|l_{max}|$=4) and thus makes stronger contribution to the intensity of adjacent pixels, which further strengthens the API. By contrast, there is no interference at all in the reconstructed images in the pseudo incoherent case, thus the pixels with Gaussian fundamental mode that represent the signal can always be clearly distinguished, displaying almost no crosstalk in the post-processed results (Fig. S9b).

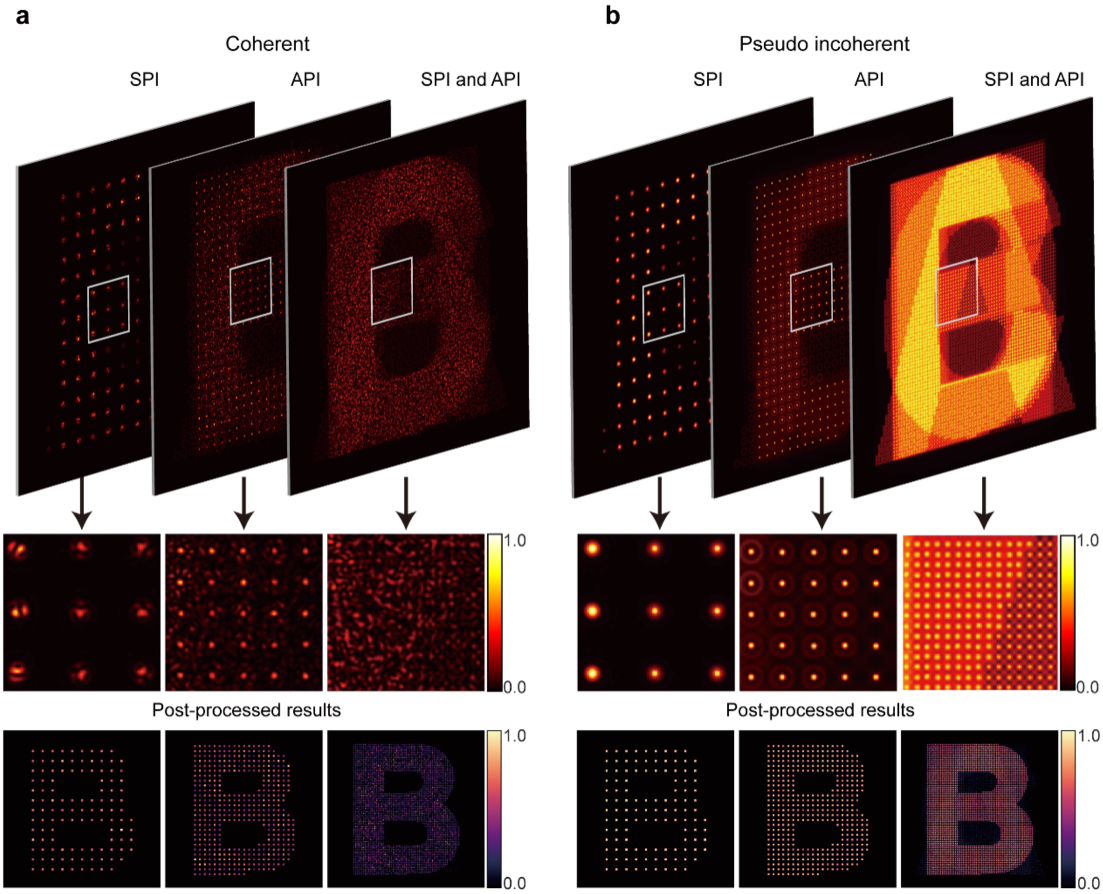

Fig. S9. Characterization of two types of interference in reconstructed images. a From left to right are the reconstructed images with SPI only, API only, and combinative effect (top row), together with enlarged views (middle row) and post-processed results (bottom row). **b** Results for the pseudo incoherent case.

Supplementary Note 9: Signal window minimum average error (SWMAE) algorithm

The error diffusion algorithm sequentially binaries each image pixel, and diffuses the binary error of current pixel to neighboring unbinarised pixels. Previous work[1] has shown that error diffusion operations on holograms results in regions of weak binary noise distribution (signal windows) in the frequency domain, while the location and shape of the signal windows are related to the error diffusion coefficient. In the process of generating Fourier holograms, high quality reconstruction can be obtained by placing the image in a signal window. However, conventional error diffusion algorithms can only produce a small signal window, which is not sufficient for high bandwidth displays. In addition, those algorithms cannot control the shape and size of signal windows by the diffusion coefficient design. SWMAE algorithm[2], as an improved error diffusion algorithm, directly links the reconstruction plane signal window of computer-generated hologram (CGH) to the diffusion coefficient.

Figure S10a illustrates the basic principle of SWMAE algorithm. Assuming that the hologram to be quantified is a grey hologram, the error $e(i,j)(i,j = 0,1,2,...,N-1)$, arising from the quantization of continuously varying grayscale values $f(i,j)$ into binary values $b(i,j)$, can be written as

$$e(i,j) = f(i,j) - b(i,j) \tag{11}$$

In the SWMAE algorithm, the error of pixel under study spreads into region A, as denoted in the figure, thus the absolute quantization error ε can be written as

$$\varepsilon = f(i,j) + \sum_{r,s\in A} d(r,s)e(i-r, j-s) - b(i,j) \tag{12}$$

The diffusion coefficient is set as $d(0,0) = 1$. The absolute quantization error can be written in the form of a convolution of diffusion coefficient, and the error can be expressed as

$$\begin{aligned} \varepsilon &= f(i,j) + \sum_{r,s\in A} d(r,s)e(i-r, j-s) - b(i,j) \\ &= \sum_{r,s\in A^0} d(r,s)e(i-r, j-s) \\ &= d \otimes e \end{aligned} \tag{13}$$

where A^0 is the domain containing the pixel being quantized. Equation 13 illustrates that SWMAE algorithm serves to reduce the convolution between the diffusion coefficient and the error. The error between the desired reconstruction $F(I,J)$ and the reconstruction of the binarized hologram $B(I,J)$ is defined as

$$E^2(I,J) = [F(I,J) - B(I,J)]^2 \tag{14}$$

Here we introduce a weighting function $W(I,J)$ to distinguish the importance of errors in different regions. Generally, W is set as 1 in the signal window (Fig. S10b) and 0 elsewhere. After defining the weighting function, the total error of the reconstructed image can be written as

$$\zeta^2 = \sum_{I,J=0}^{N-1} W^2 (F-B)^2 \begin{cases} W=1 & (\mathrm{I,J}) \in \text{signal} \\ W=0 & \text{other} \end{cases} \tag{15}$$

This error can be transformed into the hologram domain by using Parseval’s theorem

$$\begin{aligned} \zeta^2 &= \sum_{i,j=0}^{N-1} [w \otimes (f-b)]^2 \\ &= \sum_{i,j=0}^{N-1} [w \otimes e]^2 \end{aligned} \tag{16}$$

Comparing Equations 13 and 16, the sum of binary errors on the hologram is actually equal to the sum of the binary quantization noise of the reconstruction, when we set

$$d(i, j) = w(i, j) \tag{17}$$

where *w* is the Fourier spectrum of the weighting function *W*. Consequently, the SWMAE algorithm essentially reduces the binary quantization noise of the signal window in the holographic reconstruction plane.

In summary, the SWMAE algorithm efficiently sets the diffusion coefficient *d* to the Fourier transform of the signal window function *W*, so that the binary noise within the signal window in the reconstruction plane is significantly suppressed. Figure S10c shows the binary hologram after the SWMAE algorithm transformation, while Figs. S10d and S10e show the reconstruction results located within and outside the predefined signal region, respectively.

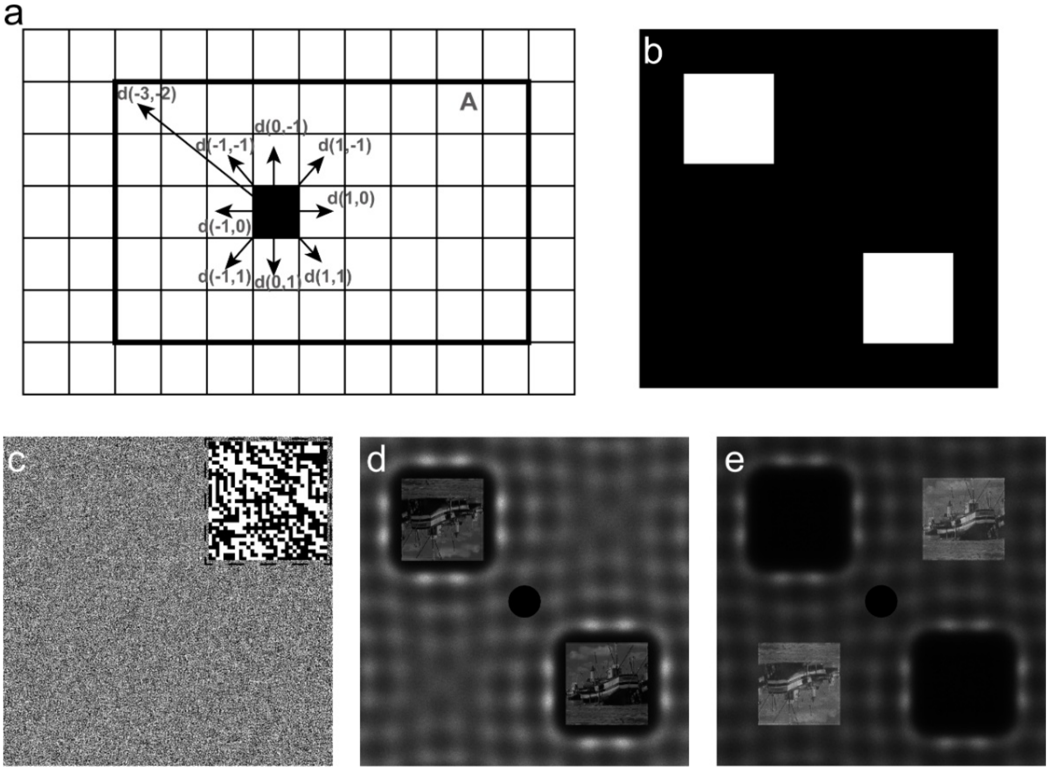

Fig. S10 Schematic diagram of SWMAE algorithm and the corresponding reconstruction results. a Schematic diagram of error diffusion and diffusion region. **b** Specified signal window in the frequency domain (Signal window weighting function). **c** Binary hologram generated using

SWMAE algorithm. **d** Simulated reconstruction of a binary hologram with the reconstructed image located within the signal window. **e** Simulated reconstruction of a binary hologram with the reconstructed image located outside the signal window.

Supplementary Note 10: Reconstruction quality improvement by temporal multiplexing

To illustrate in detail the process of TMBH, a reconstruction example is demonstrated in Fig. S11, in which five densely sampled binary images are encoded into 50 binary OAM multiplexed holograms using five helical phase functions with Δl=1. We arrange a series of binary OAM multiplexed holograms in temporal sequence (T_1~T_{50}, see Fig. S11a), and the reconstruction result at each moment is shown in Fig. S11b, which appear as an array of ambiguous speckles, since dense sampling leads to severe interference between adjacent pixels. However, after accumulating intensities of reconstructed results of all timings (T_1~T_{50}) during a modulation period, a distinctive figure visible to the detector and human eyes is obtained by TMBH method, where the enlarged view in Fig. S11b clearly exhibits the doughnut-shaped OAM mode pixels and the Gaussian mode pixels, as dictated by a solid-spot intensity distribution in the reconstructed image, leading to a remarkably high reconstruction quality, very close to that of the pseudo incoherent case. The statement is further supported by Fig. S11c that as the number of temporal superposition N_t increases, the CV value of the decoded image drops sharply from 34% at N_t=1 to 19% at N_t=10, and then slowly converges to ~17% with addition of tens of more temporal slices, which is close to the value of 15.6% for the pseudo incoherent case (red dashed line). Comparison between two insets of the decoded images at N_t=1 and N_t=50 in Fig. S11c demonstrate the significant improvement of reconstruction quality by temporal multiplexing.

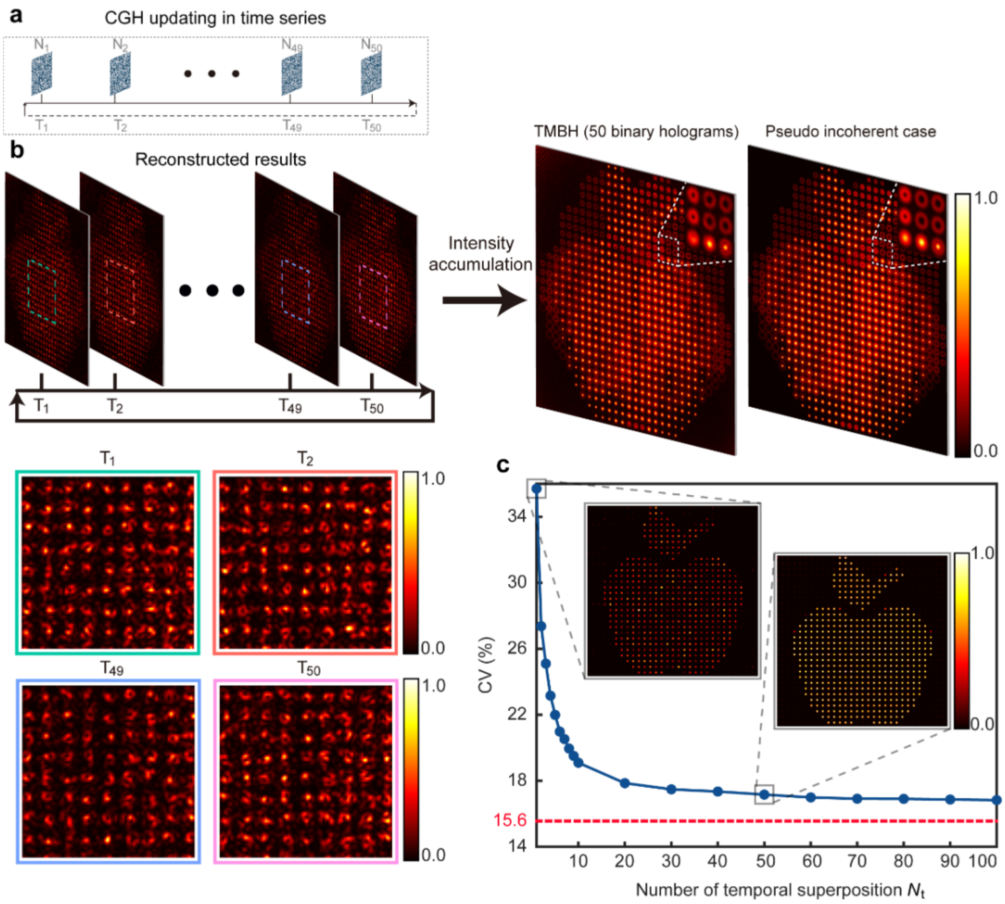

Fig. S11. Reconstructed results of temporal multiplexing binary OAM holography, compared with that of pseudo incoherent case. a Temporal multiplexing of 50 binary holograms. **b** Reconstructed results at each moment in temporal multiplexing (left). Intensity accumulation of the reconstructed results for all moments from T_1-T_{50}, compared with the reconstructed results of pseudo incoherent case (right). **c** Improvement on reconstruction quality of the decoded image using temporal multiplexing. The red dashed line denotes the CV of pseudo incoherent case.

Supplementary Note 11: Analysis of OAM content of TMBH approach

We depict Fig. S12 to illustrate an example of the reconstruction results in Fig. S11. Five binary images are encoded into a single OAM multiplexed hologram through five independent OAM channels (Fig. S12a). For each of the five images, an OAM-preserved hologram is designed, and then the OAM channels are distinguished by five encoding helical phase plates (l=2, 1, 0, -1, -2). The image encoded in each OAM channel can be decoded by an OAM beam having a helical phase index opposite to that of the corresponding OAM channel. In the example of Fig. S11, the OAM beam with l=2 is used to decode an image with the content of an apple (Fig. S12b), and the decoding results of T_1, T_2, T_{49}, T_{50} and the decoding results of TMBH (combining 50 binary holograms) are shown in Fig. S11b. On the right part of Fig. S11b, it can be seen that the pixel locations with Gaussian patterns have significantly stronger intensity distributions, and these pixel locations depict the shape of an apple, which is the basic principle of OAM beam decoding.

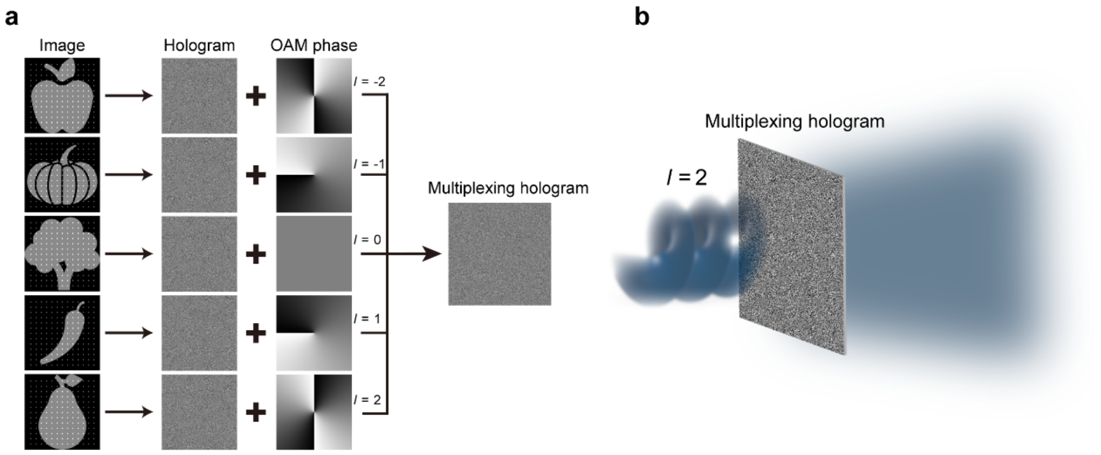

Fig. S12 Detailed explanation of the reconstruction example in Fig. S11. a Design flow chart of OAM multiplexed hologram. **b** Schematic diagram of the reconstruction example in Fig. S11.

Using the parallelized topological charge analysis[3], we obtain the OAM content at all pixels in the reconstructed image with a single measurement, with the experimental setup shown in Fig. S13. We experimentally measure the OAM content in the reconstructed images of TMBH, as shown in Fig. S14, as well as the OAM content in the reconstructed images at T_1 moment, as shown in Fig. S15. It can be noticed in Fig. S14 that the reconstructed image after temporal multiplexing contains the OAM contents of l=0,1,2,3,4. In particular, all the pixels containing l=0 form an apple pattern together (the target image), while the pixels containing l=1,2,3,4 form other patterns (images encoded into other OAM channels). The OAM content analysis on three representative pixels (p1, p2, p3) indicates that all OAM contents (l=0,1,2,3,4) are present in pixel p2, each having a near-uniform portion (~20%), and only the OAM content of l=0 is contained in

pixel p1, while pixel p3 has the OAM content of *l*=0,2, as illustrated in the bottom row of Fig. S14. In contrast, for a single moment of the reconstructed image, the content of each OAM is rather disordered, corresponding to a poorer reconstructed quality. For example, in the measurement result for *l*=0 in Fig. S15, the mode content of *l*=0 varies drastically, also the measured content of *l*=0 at pixel p2 is only 3%, far from the ideal value of around 20%.

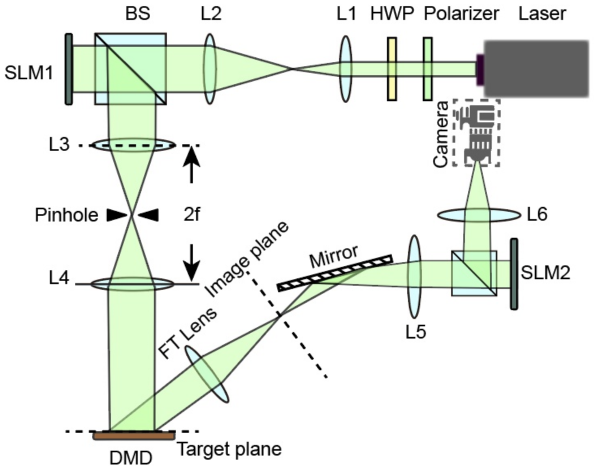

Fig. S13 Experimental setup for parallelized topological charge analysis.

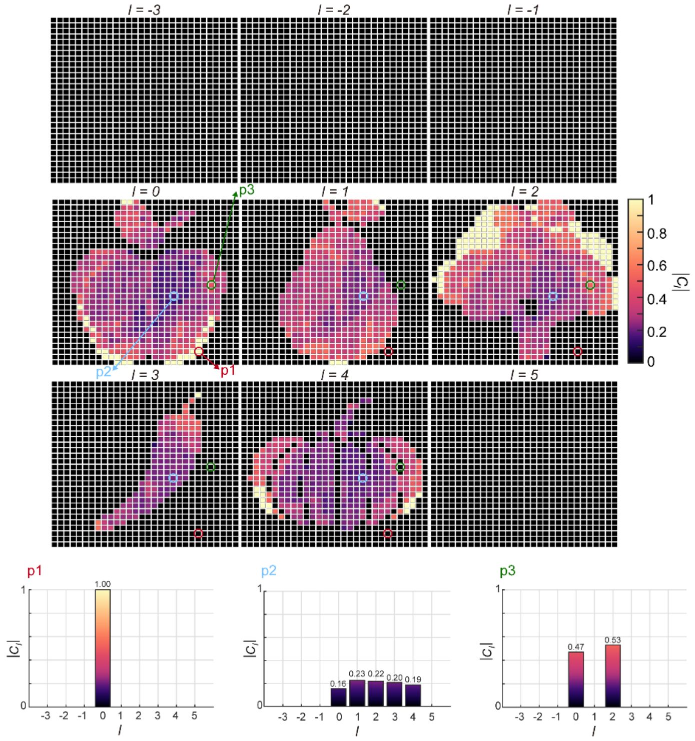

Fig. S14 Topological charge decomposition of the reconstructed image after temporal multiplexing (incident OAM beam with *l*=2) .

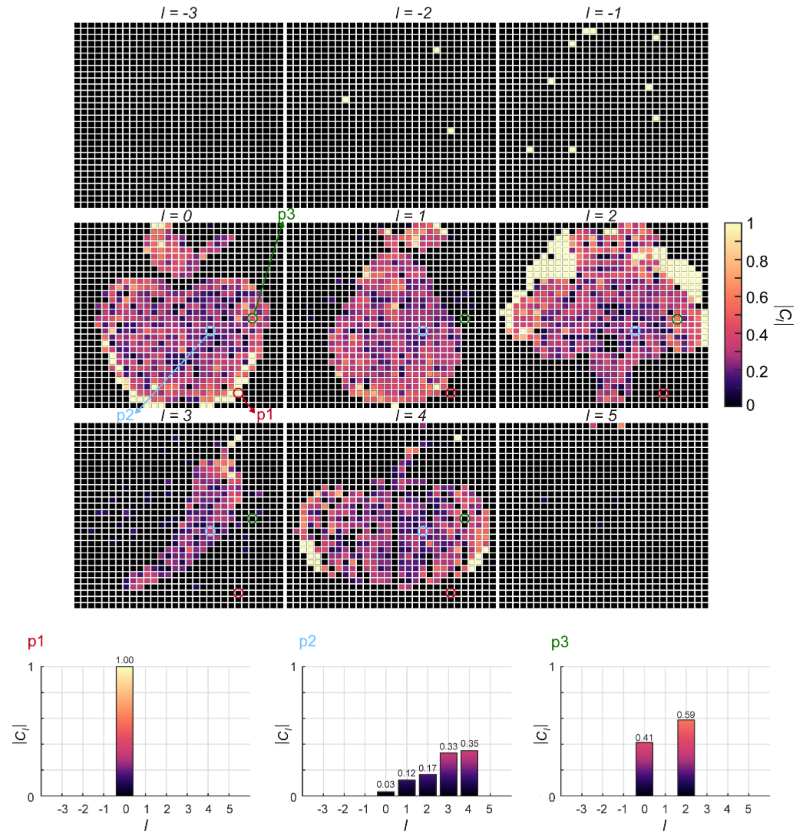

Fig. S15 Topological charge decomposition of reconstructed image at T_1 moment (incident OAM beam with *l*=2).

Supplementary Note 12: Experimental setup of TMBH approach

In the OAM holographic display setup for implementing TMBH method (Fig. S16a), we firstly use a reflective phase-only spatial light modulator (SLM, using HOLOEYE PLUTO-2-VIS-014 model with a resolution of 1920×1080 pixels and a pitch of 8 μm) to generate OAM beams, and then a high-speed digital micromirror device (DMD, using DLP6500FYE model with a resolution of 1920×1080 pixels and a micromirror pitch of 7.56 μm) to time-sequentially upload binary holograms. The beam from a solid-state laser source (CNI laser, MGL-III-532nm) is expanded to a near-plane wave by passing through a telescope (L1, focal length of 25 mm; L2, focal length of 300 mm) with the magnification of 1:12. OAM beams are conveyed from the SLM plane to the DMD plane via a 4f system with L3 and L4, both with the focal length of 200 mm. The reconstructed images in the back focal plane of the Fourier lens (focal length of 400 mm) are captured directly by a camera (Nikon D7500).

As a baseline comparison, we also built an OAM holographic display setup for phase-only OAM holography (PH) method, using two SLMs to generate OAM beams and load phase-only OAM holograms respectively (Fig. S16b). Both SLMs are of the same type (HOLOEYE PLUTO-2-VIS-014 reflective phase-only SLM with a resolution of 1920×1080 pixels and a pitch of 8 μm). All other components are identical to the TMBH setup.

Besides, we have established an OAM holographic display setup for CAH method as the baseline comparison (Fig. S16c). Different from the experimental setup of PH, two SLMs (both with a resolution of 1920×1080 pixels and a pitch of 8 μm) are used in CAH setup to load a

complex-amplitude hologram, in which the SLM2 (amplitude-only one) and SLM3 (phase-only one) are combined into a single complex amplitude modulation device through a 4f system (L5 and L6). All other components are identical to the PH setup.

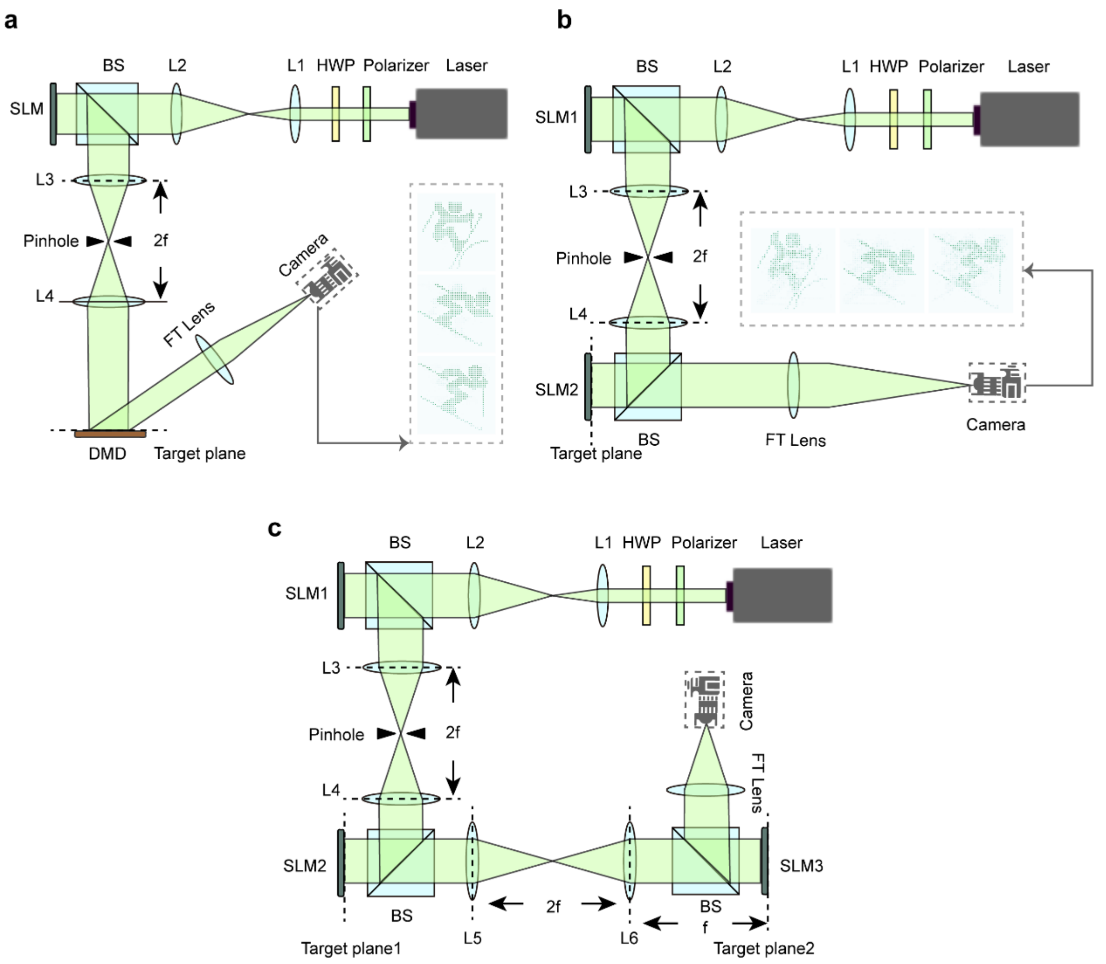

Fig. S16. Experimental setup. a DMD-based OAM holographic display prototype. **b** OAM holographic display prototype using phase-only SLM (Baseline). **c** Complex amplitude modulation using an amplitude-only SLM and a phase-only SLM (Baseline).

Supplementary Note 13: Experimental reconstruction results of binary images (γ~2)

In Fig. S17 we compare in detail the experimental performance of three types of OAM holograms: PH, CAH, and TMBH. The target images in Fig. S17 are identical to that of the simulation in Fig. 6 in the main text, i.e., a set of 300×300 binary images of winter sport icons are separately encoded into multiple OAM channels with a sampling distance of L = 8λ/ NA, and the index interval is Δl=1. Figures S17a and S17b show the reconstruction results for 7 and 11 OAM channels, respectively, indicating that the TMBH method obviously has the best image quality and the lowest CV of all OAM channels (see also Fig. S17c). Figure S17d shows that TMBH has a CV value of 8.1% at the conventional boundary γ=1 (with 7 channels), which is close to the result in the simulation (5.5%), while TMBH has a CV value of 10.1% at the extended range of γ=2 (with 11 channels), which is close to the simulated value (6.5%).

It is worth noting that the reconstruction quality of CAH in the experiment is close to PH, significantly lower than the simulated results, due to two reasons: (1) Theoretical complex

amplitude value for CAH is used in the simulation, which is an ideal value and does not take into account the quantization error. The reason for using the theoretical complex amplitude values in the simulation is to highlight the superiority of TMBH, showing that the reconstruction quality of TMBH is even better than that of the theoretical complex amplitude holography. (2) The CAH reconstruction method by using two spatial light modulators (an amplitude-type one and a phase-type one, with the experimental setup shown in Fig. S16c) is not as effective as using complex amplitude metasurfaces for complex amplitude modulation.

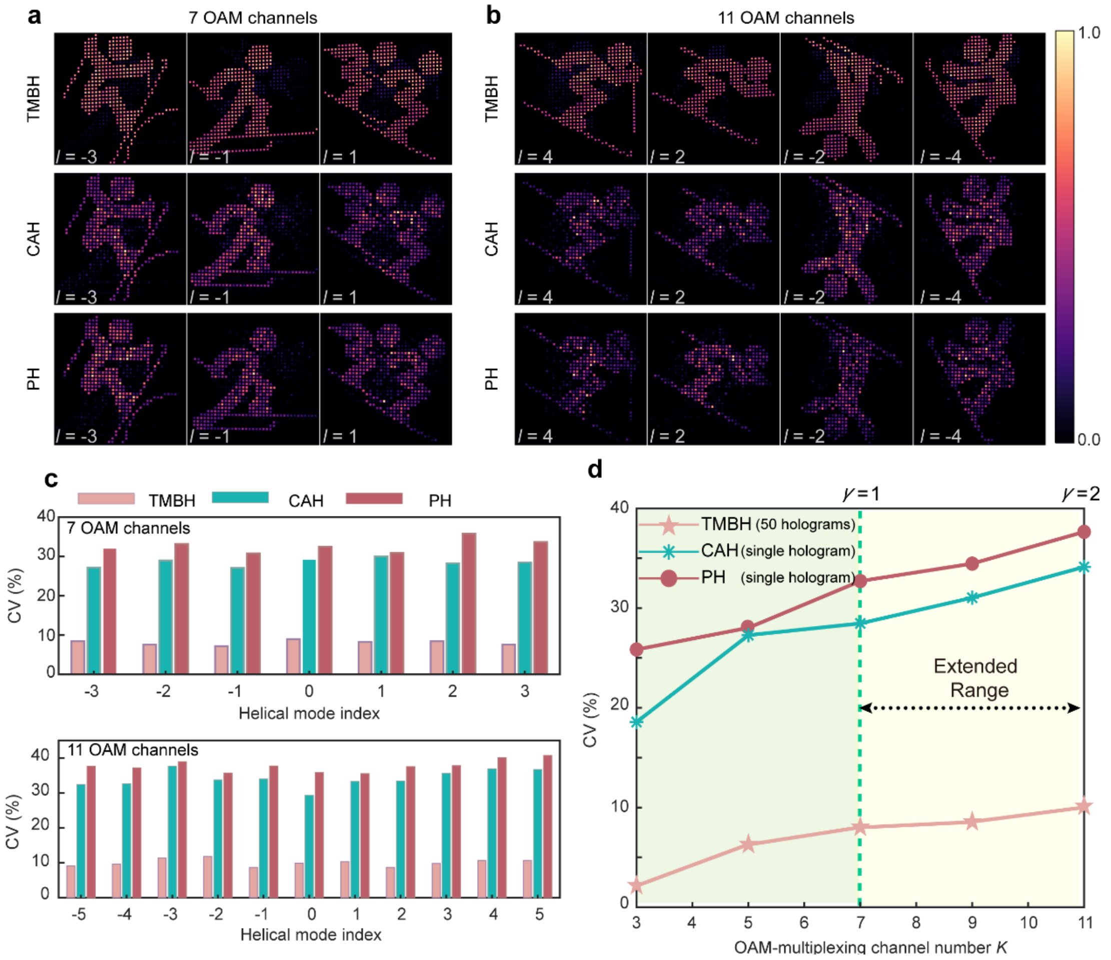

Fig. S17 Experimental reconstruction results of binary images by three classes of OAM holography. **a** Reconstructed results (partially shown) with 7 OAM channels. **b** Reconstructed results (partially shown) with 11 OAM channels. **c** Quantitative comparison of reconstruction quality. **d** Evolution of intensity fluctuation of reconstructed images versus the number of OAM multiplexing channels, where the multiplexing channel numbers for γ=1 and γ=2 are marked.

Supplementary Note 14: Experimental reconstruction results of binary images (γ~5.6)

The experimental results in Fig. S18 corresponds to the numerical results in Fig. 7 in the main text. Due to the resolution limitation of the modulation device used in the experiment (device resolution is 1080×1080, lower than the simulation resolution of 2160×2160), the resolution of the binary image used in the experiment is 400×400. Figure S18a demonstrates the capacity scaling capability at a fixed resolution (L = 10 λ/NA) that as the number of channels K increases from 5 to 45, the TMBH method can always reconstruct the multiplexed images, having the CV value increased from 9.5% to 29.0%. In contrast, CAH method fails to recover the object at large value of K, with the CV dramatically increased from 35.8% to 83.3%. The experimental reconstruction of TMBH method exceeds the conventional resolution limit of OAM holography, showing a 5.6-fold resolution improvement (from L=56 λ/NA to L=10 λ/NA).

Note that the experimental results of TMBH presented in Fig. S18 are inferior to the simulation results presented in Fig. 7 in two aspects: (1) The fluctuation of the reconstructed images in Fig. S18a (CV increases from 9.5% to 29.0% as K increases from 5 to 45) is higher than that in Fig. 7a (CV increases from 7.6% to 17.6% as K increases from 11 to 81). (2) The background noise of the reconstructed images in Figure S18b (top row) is stronger than that in Fig. 7b (top row). There are three main reasons why the experimental results have lower quality than the simulation results: (1) Each of multiplexed channels in the experiment of OAM holography takes a portion of incident intensity, thus multiplexing up to dozens of images demands a rather strong incident beam, otherwise the signal from single channel is too weak for camera acquisition. However, the increasing level of incident intensity inevitably aggravates SPI and API effects in the reconstructed images. (2) The final reconstructed images shown are obtained after the filtering aperture array in post-processing, while the filtering aperture array is calculated from the captured spot array. However, due to calculation error, the spatial location of the calculated filter aperture array may be deviated from the actual location by a few pixels, allowing light from undesirable OAM channels enter the filter aperture. This effect increases the intensity fluctuation in the experimentally reconstructed image compared with simulation, especially when multiplexing a lot of images since the light from the other OAM channels is much stronger. (3) The experimental results are inevitably affected by the modulation error of the modulation device.

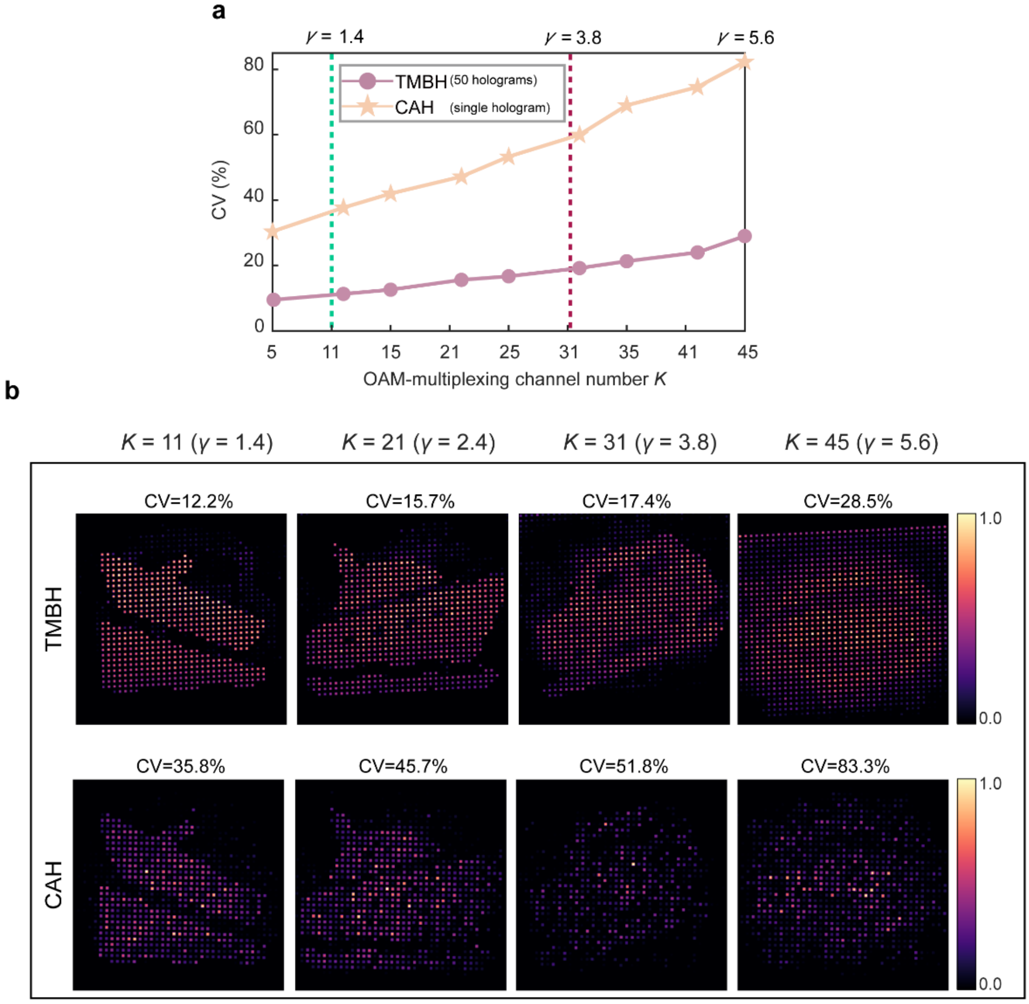

Fig. S18 Super-resolution reconstruction of binary images by TMBH method in experiments. **a** Intensity fluctuations of reconstructed images versus increasing OAM-multiplexing channel number. **b** Comparison between TMBH and CAH methods in terms of reconstruction quality at same resolution, operating far beyond the sampling criterion limit.

Supplementary Note 15: Demonstration of TMBH in three-dimensional (3D) holography

Here we illustrate by two examples that TMBH can be applied in 3D holography. The first example is a 3D OAM-selective hologram[4], with the reconstructed results by TMBH method shown in Fig. S19. In the plane of z=400 mm, an incident OAM beam with helical mode index l=1 can reconstruct the image of the Big Ben, while the other image (the Eiffel Tower) is out of focus in this plane. In the plane of z=450 mm, an incident OAM beam with helical mode index l=2 reconstructs the image of the Eiffel Tower, while the image of the Big Ben is out of focus.

The second example is a 3D OAM-multiplexing hologram[5], illuminated sequentially by OAM beams with helical mode indices ranging from -4 to 4, resulting in a total of 10 images reconstructed in two different planes, as shown in Fig. S20.

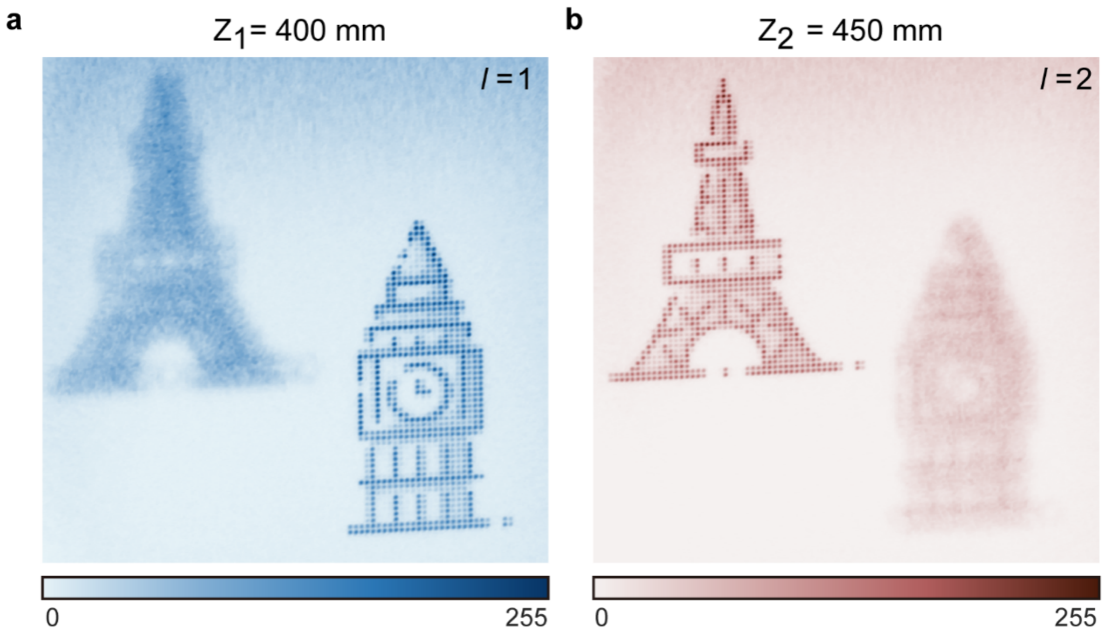

Fig. S19 Experimental demonstration of using an OAM-selective hologram for the reconstruction of three-dimensional OAM-dependent holographic images. a The experimentally reconstructed holographic images using an incident OAM beam with a helical mode index of l = 1. **b** The experimentally reconstructed holographic images using an incident OAM beam with a helical mode index of l = 2.

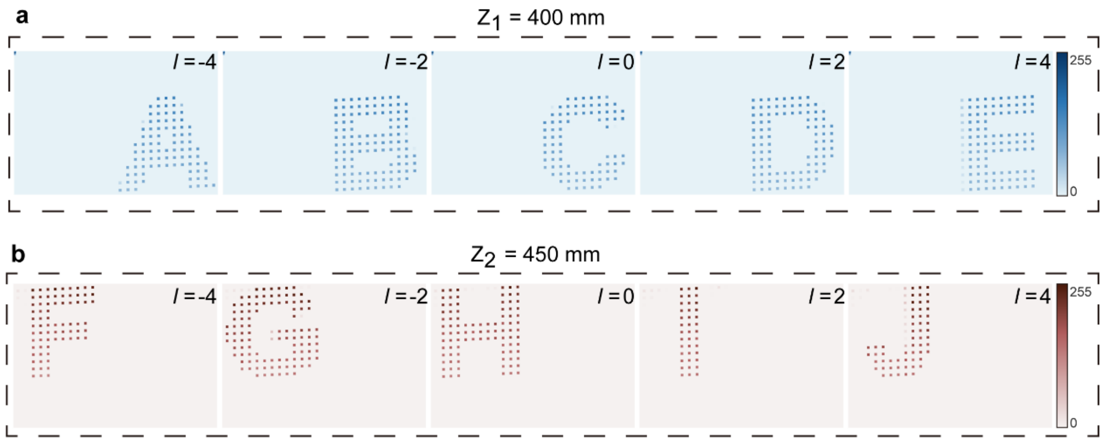

Fig. S20 Experimental demonstration of using an OAM-multiplexing hologram for the reconstruction of three-dimensional OAM-dependent holographic images. a Reconstructed images from an OAM-multiplexing hologram at a reconstruction distance of z=400 mm. **b** Reconstructed images from an OAM-multiplexing hologram at a reconstruction distance of z=450 mm.

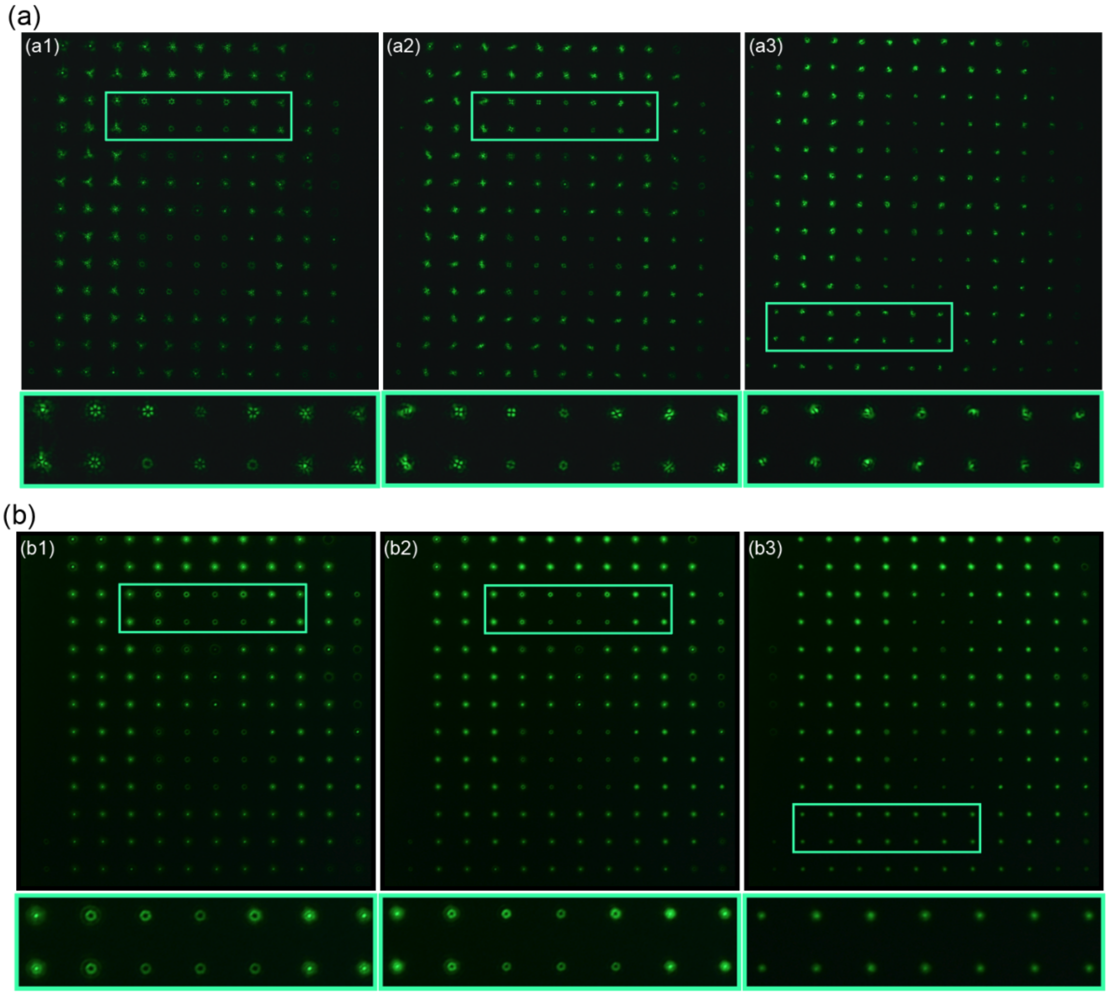

Fig. S21. Superposed mode pixels and OAM mode pixels in the reconstructed image. a Superposed mode pixels in the reconstructed images of PH method, including reconstructed results by 5 OAM channels with Δl=3 (a1) and Δl=2 (a2), and results by 7 OAM channels with Δl=*1* (a3). **b**: OAM mode pixels in the reconstructed images of TMBH method, including reconstructed results by 5 OAM channels with Δl=3 (b1) and Δl=2 (b2), and results by 7 OAM channels with Δl=1 (b3).

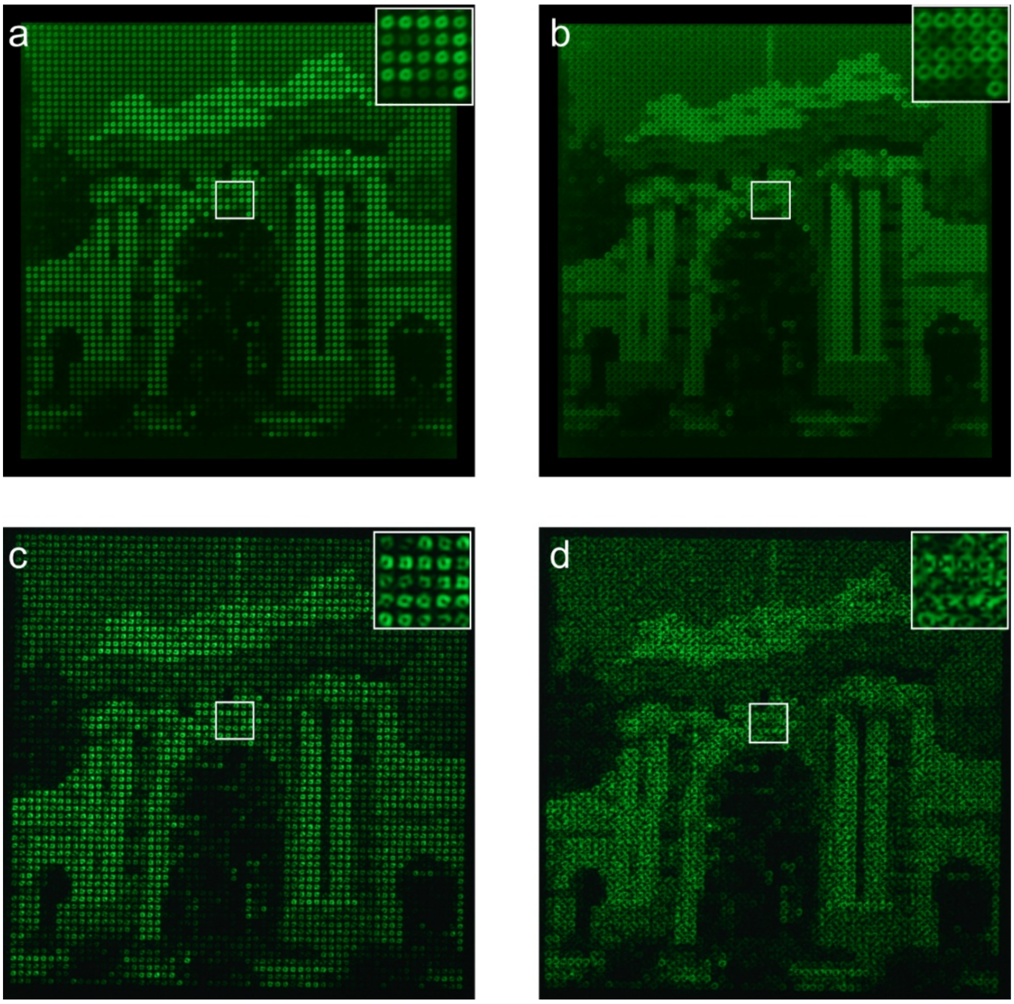

Fig. S22. Experimental results for TMBH and PH holograms. a-b Reconstructed images of TMBH method, using incident OAM beams with l = 1 (a) and l = 2 (b). **c-d** Reconstructed images of PH method, using incident beams with l = 1 (c) and l = 2 (d).

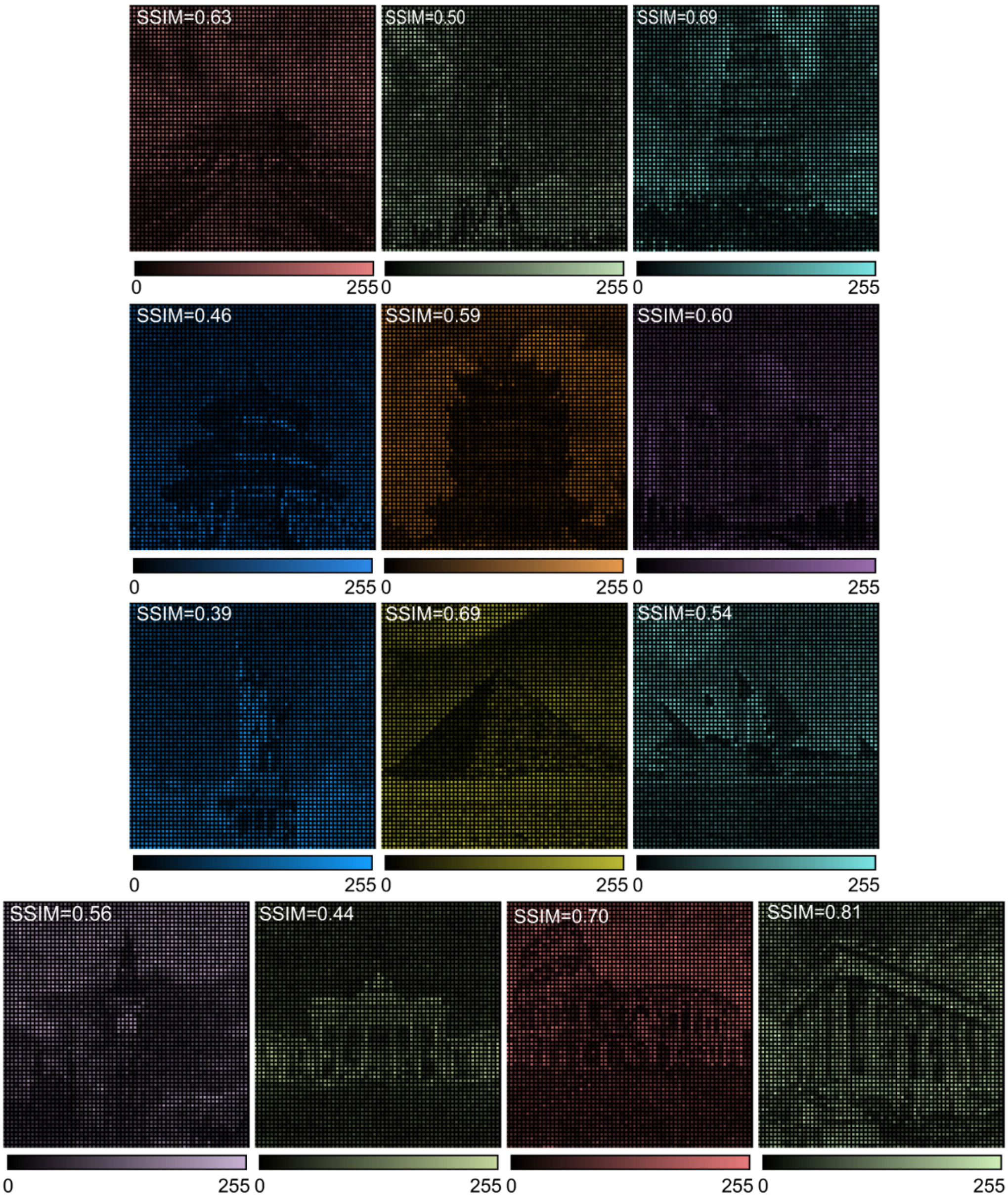

Fig. S23 Simulation results of densely sampled grayscale reconstructed images by TMBH method. The SSIM values of reconstructed images using TMBH with 13 OAM channels here are comparable to those using CAH with 5 OAM channels (Fig. 8 in the main text). The images of famous landmarks from left to right and from top to bottom are: 'Forbidden City', 'Oriental Pearl Tower', 'Giant Wild Goose Pagoda', 'Temple of Heaven', 'Yellow Crane Tower', 'Taj Mahal', 'Statue of Liberty', 'Pyramids', 'Sydney Opera House', 'Big Ben', 'Brandenburg Gate', ' Roman Colosseum', and 'Parthenon'.

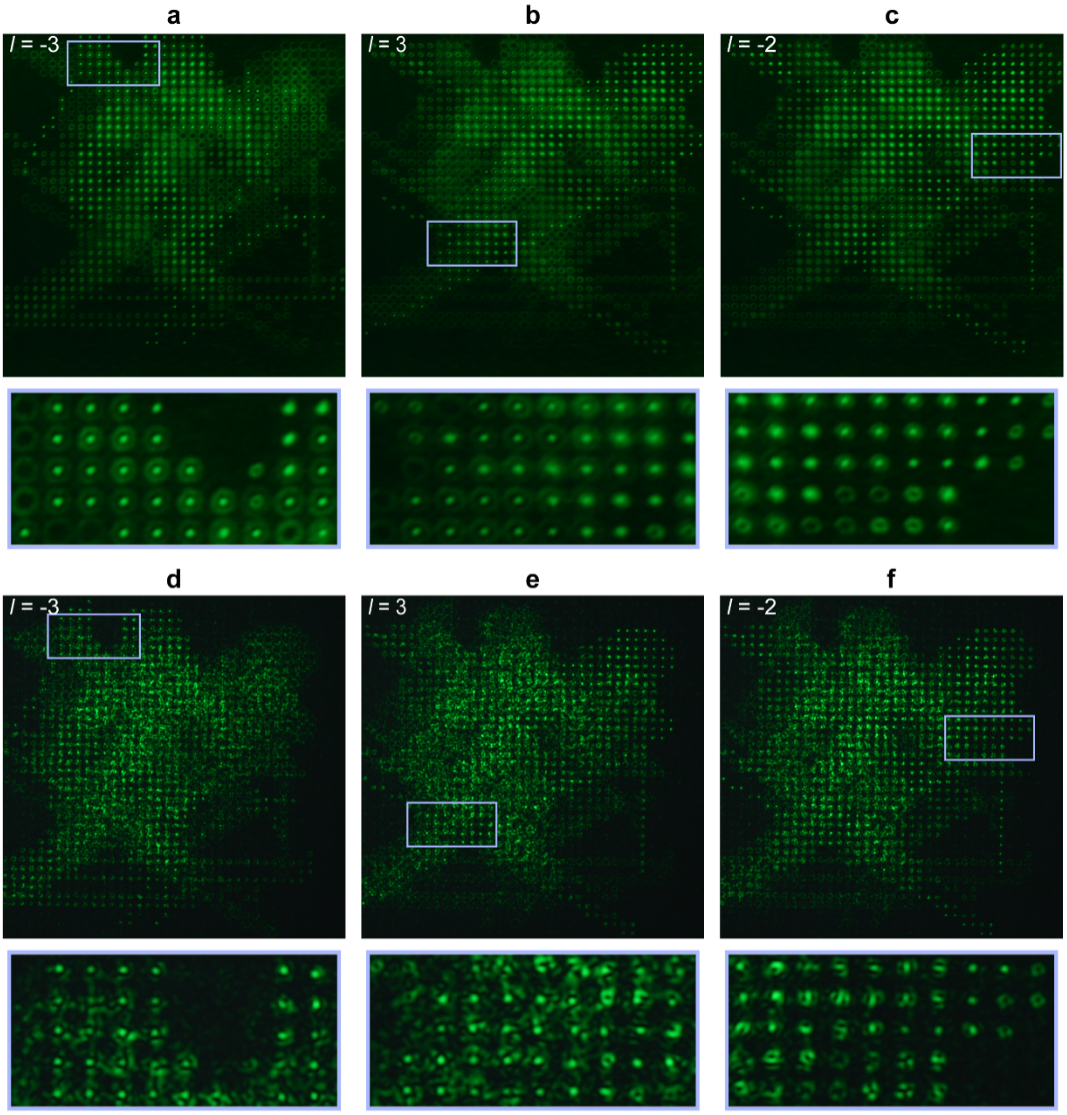

Fig. S24. Raw results of partial reconstructed images of winter sport icons from OAM holograms carrying 7 OAM channels, taken directly by camera. a-c: TMBH method. **d-f**: PH method.

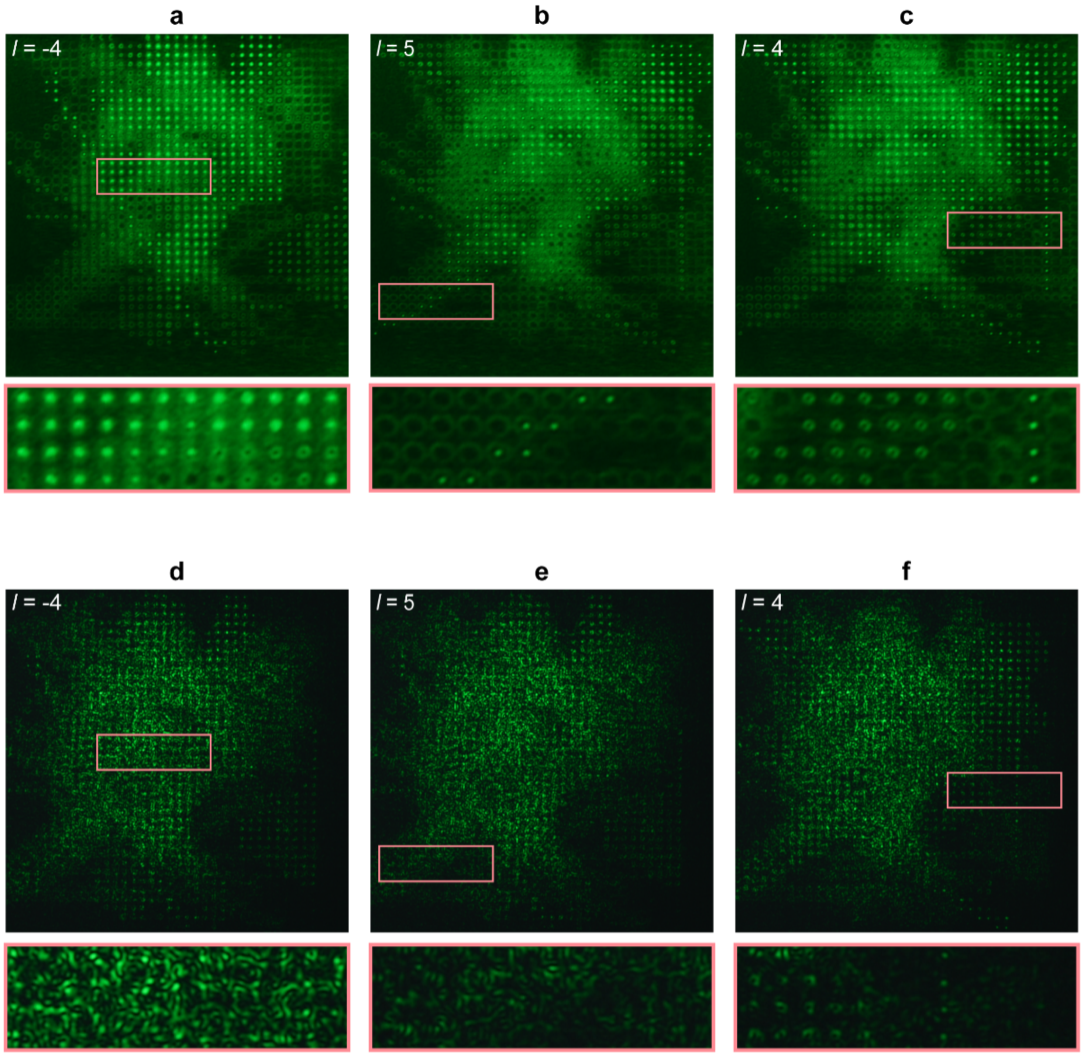

Fig. S25. Raw results of partial reconstructed images of winter sport icons from OAM holograms carrying 11 OAM channels, taken directly by camera. a-c: TMBH method. **d-f**: PH method.

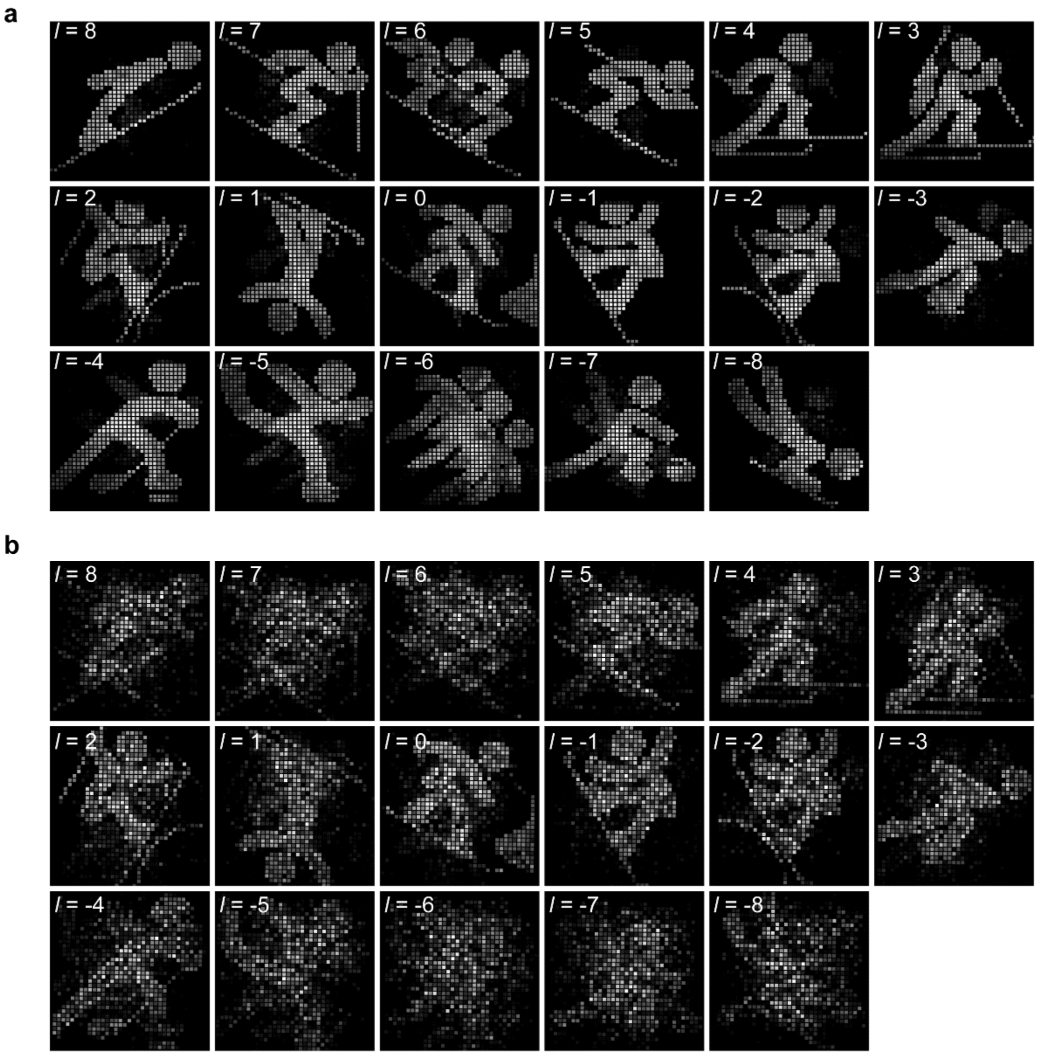

Fig. S26. Comparison of reconstructed results with γ= 2.5 and 17 OAM-multiplexing channels. a TMBH method. **b** PH method.

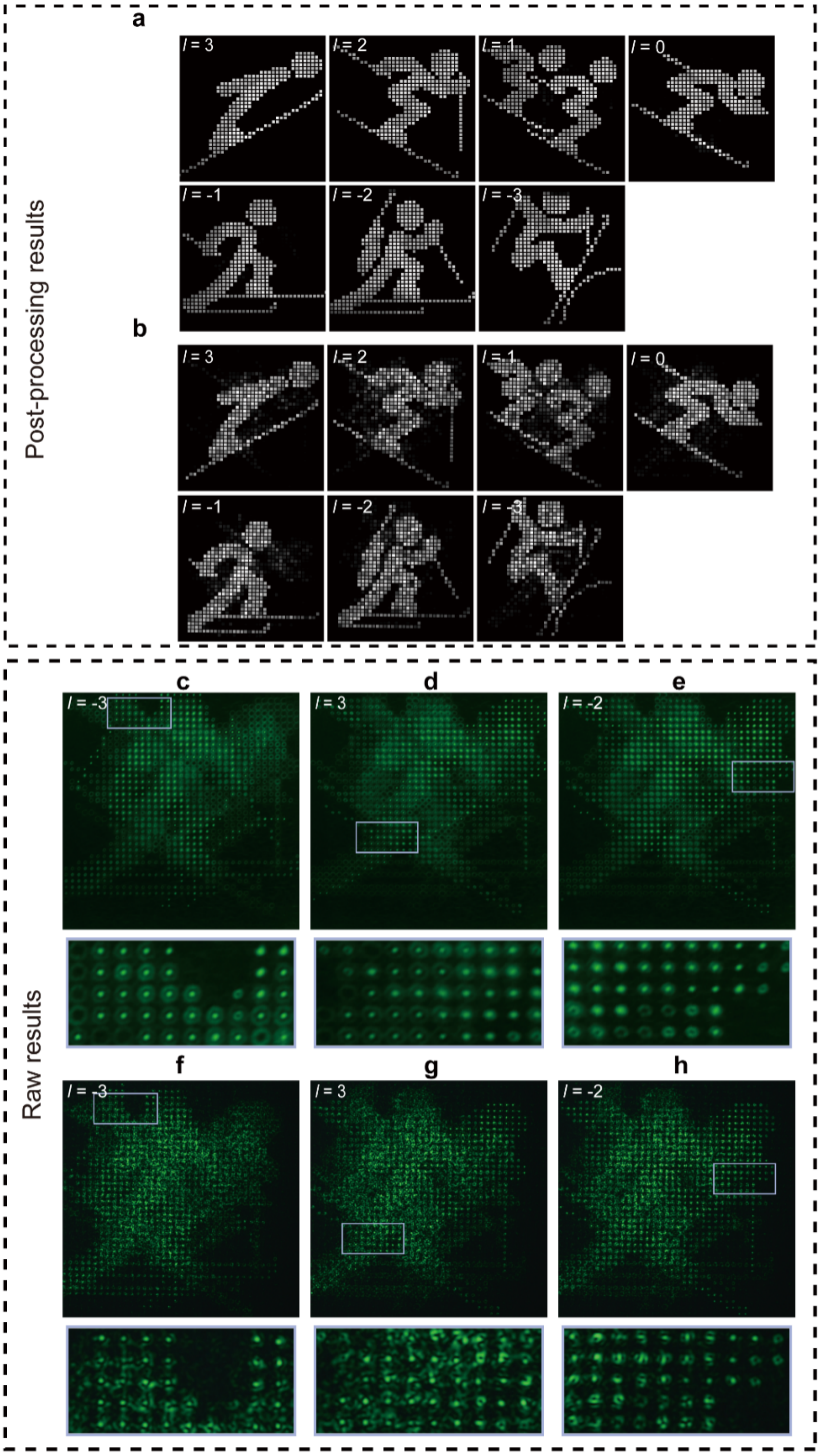

Fig. S27. Post-processing results and raw results of the reconstructed binary images of winter sport icons from holograms carrying 7 OAM channels. a TMBH method (post-processed). **b** PH method (post-processed)**. c-e**: TMBH method (raw). **f-h**: PH method (raw).

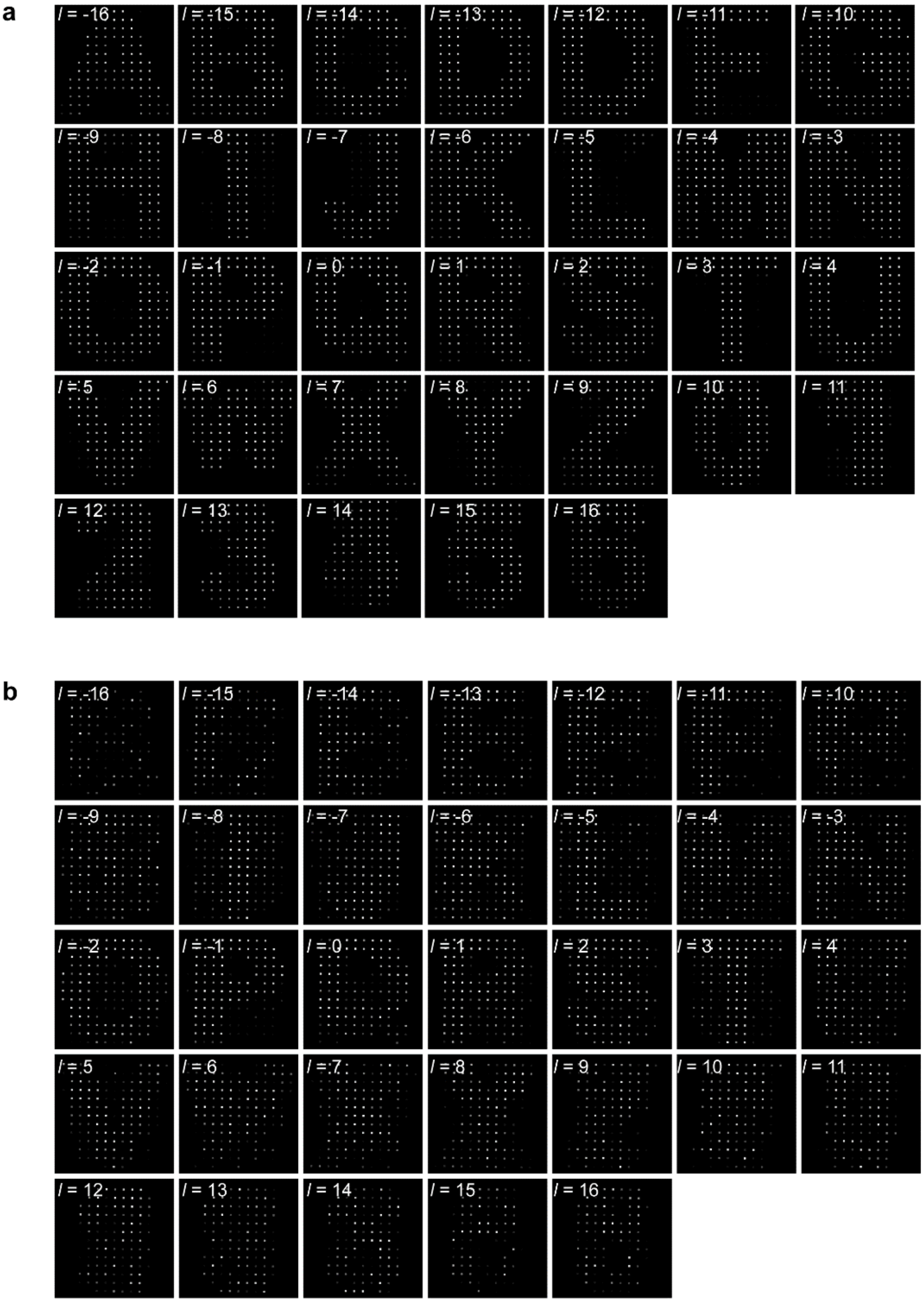

Fig. S28. Comparison of 33 reconstructed images of sparsely sampled letters and numbers. a TMBH method. **b** PH method.

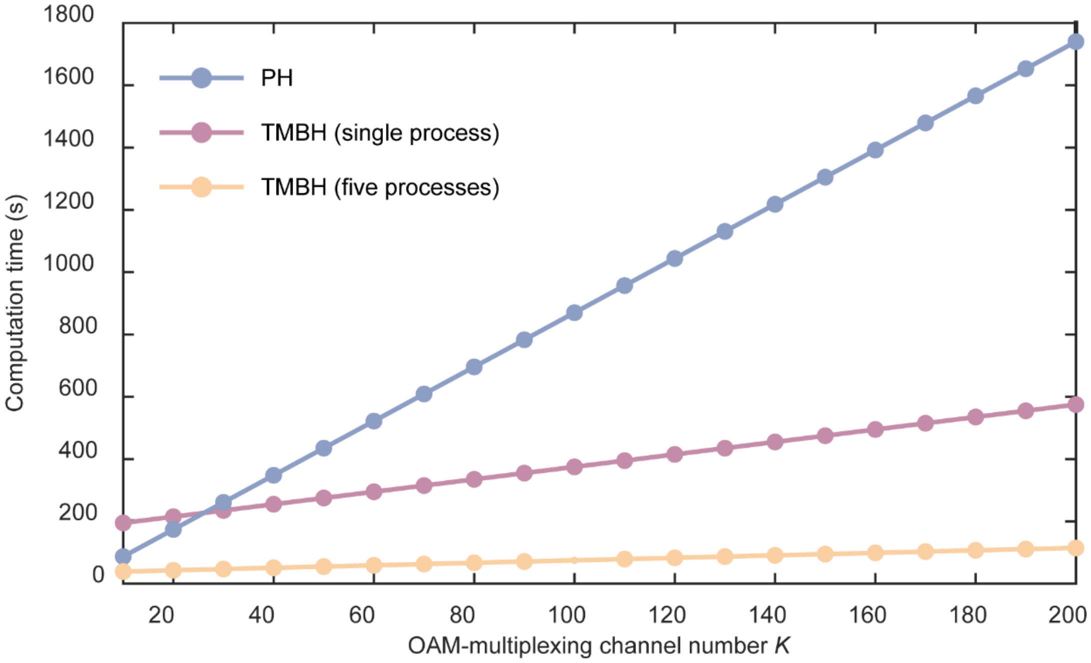

Fig. S29. Comparison of the computation times for TMBH method with 50 holograms versus PH method with 1 hologram. The computation time using five computer processes simultaneously is marked as well (orange).

References

1. Weissbach, S. & Wyrowski, F. Error diffusion procedure: theory and applications in optical signal processing. *Appl. Opt.* **31**, 2518-2534 (1992).
2. Heggarty, K. & Chevallier, R. Signal window minimum average error algorithm for computer-generated holograms. *J. Opt. Soc. Am. A* **15**, 625-635 (1998).
3. Sroor, H. et al. High-purity orbital angular momentum states from a visible metasurface laser. *Nat. Photonics* **14**, 498-503 (2020).
4. Fang, X., Ren, H., & Gu, M. Orbital angular momentum holography for high-security encryption. *Nat. Photonics* **14**, 102-108 (2020).
5. Ren, H. et al. Complex-amplitude metasurface-based orbital angular momentum holography in momentum space. Nat. Nanotechnol. **15**, 948-955 (2020).
