# Supplementary material for: Super-resolution orbital angular momentum holography
Source: Nat Commun. 2023 Apr 4;14:1869. doi: 10.1038/s41467-023-37594-7 (PMC10073211; doi:10.1038/s41467-023-37594-7)
Supplement: Supplementary file 3 — Description of Additional Supplementary Files [file 41467_2023_37594_MOESM3_ESM.pdf]

**Description of Additional Supplementary Files**

File Name: Supplementary Movie 1
Description: OAM holographic video display reconstructed from 201-OAM-channel-multiplexing hologram by TMBH method

File Name: Supplementary Movie 2
Description: OAM holographic video display reconstructed from 201-OAM-channel-multiplexing hologram by CAH method
